# Genomic organization and splicing evolution of the *doublesex *gene, a *Drosophila *regulator of sexual differentiation, in the dengue and yellow fever mosquito *Aedes aegypti*

**DOI:** 10.1186/1471-2148-11-41

**Published:** 2011-02-10

**Authors:** Marco Salvemini, Umberto Mauro, Fabrizio Lombardo, Andreina Milano, Vincenzo Zazzaro, Bruno Arcà, Lino C Polito, Giuseppe Saccone

**Affiliations:** 1 Department of Biological Sciences - Section of Genetics and Molecular Biology. University of Naples "Federico II" - Italy; 2 CDF - Centro Diagnostico Flegreo, Naples - Italy; 3 Department of Public Health - Parasitology Section. University of Rome "La Sapienza" - Italy; 4 Department of Structural and Functional Biology. University of Naples "Federico II" - Italy; 5 Institute of Genetics and Biophysics "Adriano Buzzati-Traverso" CNR Naples - Italy

## Abstract

**Background:**

In the model system *Drosophila melanogaster, doublesex *(*dsx*) is the double-switch gene at the bottom of the somatic sex determination cascade that determines the differentiation of sexually dimorphic traits. Homologues of *dsx *are functionally conserved in various dipteran species, including the malaria vector *Anopheles gambiae*. They show a striking conservation of sex-specific regulation, based on alternative splicing, and of the encoded sex-specific proteins, which are transcriptional regulators of downstream terminal genes that influence sexual differentiation of cells, tissues and organs.

**Results:**

In this work, we report on the molecular characterization of the *dsx *homologue in the dengue and yellow fever vector *Aedes aegypti *(*Aeadsx*). *Aeadsx *produces sex-specific transcripts by alternative splicing, which encode isoforms with a high degree of identity to *Anopheles gambiae *and *Drosophila melanogaster *homologues. Interestingly, *Aeadsx *produces an additional novel female-specific splicing variant. Genomic comparative analyses between the *Aedes *and *Anopheles dsx *genes revealed a partial conservation of the exon organization and extensive divergence in the intron lengths. An expression analysis showed that *Aeadsx *transcripts were present from early stages of development and that sex-specific regulation starts at least from late larval stages. The analysis of the female-specific untranslated region (UTR) led to the identification of putative regulatory *cis*-elements potentially involved in the sex-specific splicing regulation. The *Aedes dsx *sex-specific splicing regulation seems to be more complex with the respect of other dipteran species, suggesting slightly novel evolutionary trajectories for its regulation and hence for the recruitment of upstream splicing regulators.

**Conclusions:**

This study led to uncover the molecular evolution of *Aedes aegypti dsx *splicing regulation with the respect of the more closely related Culicidae *Anopheles gambiae *orthologue. In *Aedes aegypti*, the *dsx *gene is sex-specifically regulated and encodes two female-specific and one male-specific isoforms, all sharing a *doublesex*/*mab-3 *(DM) domain-containing N-terminus and different C-termini. The sex-specific regulation is based on a combination of exon skipping, 5' alternative splice site choice and, most likely, alternative polyadenylation. Interestingly, when the *Aeadsx *gene is compared to the *Anopheles dsx *ortholog, there are differences in the *in silico *predicted default and regulated sex-specific splicing events, which suggests that the upstream regulators either are different or act in a slightly different manner. Furthermore, this study is a premise for the future development of transgenic sexing strains in mosquitoes useful for sterile insect technique (SIT) programs.

## Background

DSX proteins are part of the Dmrt (*doublesex *and *mab-3*-related transcription factor) family, a structurally and functionally conserved group of zinc-finger proteins with relevant roles in sex determination and sexual differentiation throughout the animal kingdom [1,2].

In *Drosophila melanogaster *and many other dipteran species, *dsx *orthologues produce sex-specific transcripts through alternative splicing, which encode two highly conserved isoforms that share a common N-terminus containing a zinc-finger domain (named DM domain) [3]. The DSX sex-specific isoforms are responsible for the proper sexual differentiation of somatic tissues and the gonads [4-7]. The female-specific splicing of the *dsx *pre-mRNA is under the control of the conserved Transformer (which is female-specifically expressed) and Transformer-2 (a non-sex-specific auxiliary factor) splicing regulators in *Drosophila *and other dipteran species, such as *Ceratitis capitata *[8,9] and other Tephritidae species [10,11], *Musca domestica *(Muscidae) [12,13] and *Lucilia cuprina *(Calliphoridae) [14]. Sequence comparisons led to the identification of key splicing regulatory elements, the so-called TRA/TRA-2 binding sites, conserved in different *Drosophila *species and in the female-specific exon of these *dsx *orthologous genes from non-Drosophilidae families. In addition to the sex-specific regulation, the functions exerted during sexual development by the two DSX isoforms are evolutionarily conserved. For example, ectopic expression of either the male-specific or the female-specific isoform of *Musca domestica *(MdDSX^M ^and MdDSX^F^) [15], *Ceratitis capitata *(CcDSX^M^) [16] and *Anastrepha obliqua *(AoDSX^M ^and AoDSX^M^) [17] into *Drosophila *transgenic flies caused a partial masculinization of XX and a partial feminization of XY individuals, respectively.

In the mosquito *Anopheles gambiae *(Diptera, Culicidae), a *dsx *ortholog was previously isolated, and it maintains sex-specific regulation by alternative splicing and putative TRA/TRA-2 binding sites in the female-specific exon [18,19]. However, despite the availability of a genome sequence, it is still unclear whether *dsx *is also under the control of the TRA-related and TRA-2 orthologous proteins in this species, as in the *Drosophila*, Tephritidae, Muscidae and Calliphoridae species.

Outside the order Diptera, *dsx *orthologues have been isolated in the lepidopteran *Bombyx mori *(*Bmdsx*) [20] and in the hymenopteran honeybee *Apis mellifera *(*Amdsx*) [21,22] and the parasitic wasp *Nasonia vitripennis *(*Nvdsx*) [23]. In these species, only a partial conservation of dipteran *dsx *features was reported, with sex-specific alternative splicing conserved, and different *cis*-acting elements identified. In *Bombyx mori, dsx *plays an essential role in silkworm sexual development and, when compared to *Drosophila*, interestingly shows a reversed pattern of default versus regulated sex-specific splicing [24] (for a reviews see [3]). Sex-specific splicing of the honeybee *Apis mellifera doublesex *gene revealed 300 million years of conservation at the bottom of the insect sex-determination pathway, confirming its key role in sexual differentiation [21,22]. Finally, in the other hymenopteran, *Nasonia vitripennis*, the availability of a gynandromorphic line led to the first demonstration of a direct functional association of the *dsx *orthologue with somatic sex differentiation in Hymenoptera [23].

The mosquito *Aedes aegypti *is the most important, global arthropod vector for the yellow fever and dengue viruses. *Ae. aegypti *is considered one of the best mosquito species for laboratory culture and has been used for detailed laboratory studies in various fields [25]. Furthermore, the *Aedes *genome and transcriptome sequences have been partially determined [26]. However, the genetic control of the *Aedes *sex determination is still to be clarified. In this species, the primary signal is different from that in *D. melanogaster*, where the X-chromosome dosage controls sex differentiation [27], and *An. gambiae*, where an heteromorphic Y chromosome contains a male-determining factor(s) that dominantly induces male development by its presence in an XX/XY system [28]. In *Aedes*, as observed for other culicine mosquitoes, heteromorphic sex chromosomes are absent, and sex is controlled by an autosomal locus that carries a male-determining gene, M, acting as a dominant male determiner [29].

An *Aedes aegypti *orthologue for *Drosophila doublesex *was previously identified by an *in silico *analysis of the genome, in addition to orthologues of other *Drosophila *genes potentially involved in sex determination and sexual differentiation, such as *transformer-2*, *fruitless*, *dissatisfaction *and *intersex *[26].

Here we present the genetic and genomic characterization of the *Aedes aegypti dsx *ortholog, its sex-specific expression analysis during development and a comparative evolutionary analysis. We show that *Aedes *DSX sex-specific isoforms are produced by sex-specific alternative splicing mechanisms with slight differences compared to other species. The *Aedes dsx *gene encodes indeed a novel second female-specific DSX isoform, by an exon skipping mechanism. Interestingly different putative splicing regulatory sequences have been found within the sex-specifically regulated *Aedes dsx *region, suggesting a possible model of its splicing regulation by upstream factors. Furthermore, our study opens the possibilities to identify the downstream targets of DSX in *Aedes*, which are still not well defined even in the model system *Drosophila melanogaster*, and to identify upstream splicing regulators of the *Aedes *sex determination cascade, which have not yet been isolated possibly conserved also in the other mosquitoes *An. gambiae*, vector of malaria disease.

## Results and Discussion

### Isolation and molecular characterization of the *Aeadsx *gene

**Figure 1:**
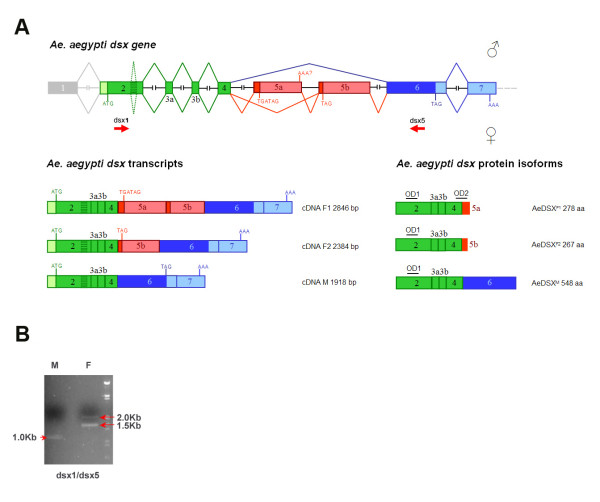
***Aeadsx *gene**. (A) Genomic organization, splicing variants and protein isoforms of *dsx *in *Aedes aegypti*. Male-specific and female-specific exons/protein regions are marked in blue and red, respectively. Exons and introns are not shown to scale. Translational start and stop sites and the poly(A) addition sites are marked. Rectangular striped box within exon 2 represents a 63-bp intronic sequence alternatively removed in *Aeadsx *transcripts of both sexes (see Figure 5B.3 for further details). All transcripts shown in this picture retain the 63-bp intronic sequence, which encodes an in-frame non-conserved 21-aa sequence. Transcripts without the 63-bp are not shown in this picture and in subsequent paper figures. (B) RT-PCR amplification of *Aeadsx *sex-specific transcripts. Primers used in this amplification are indicated as short red arrows in Figure 1A.

The molecular cloning of the *Aedes aegypti dsx *gene (*Aeadsx*) was performed by combining a classical PCR-based approach with the available bioinformatic and genomic tools. The later used the non-sex-specific region of the *Anopheles gambiae *DSX protein (236 aa) as a virtual probe for a BLASTP search of the Ensembl *Ae. aegypti *AaegL1 genomic database (http://www.ensembl.org/) (see Methods for further strategy details). A putative male-specific cDNA sequence was then obtained by EST analysis, using *An. gambiae dsx *male-specific nucleotide sequences (kindly provided by Pannuti A. and Lucchesi J., Emory University - USA, prior publication). Using specific primer pairs for the putative male-specific EST and common genomic sequences, we performed an RT-PCR analysis on RNA samples extracted from adult sexed *Ae. aegypti *mosquitoes. With this approach, we successfully amplified sex-specific products of the *Aeadsx *gene, with two female-specific products of 1.5 kb and 2.0 kb and a male-specific product of 1.0 kb (Figure 1B). cDNA products were cloned and sequenced, and their conceptual translation of incomplete ORFs and comparison with DSX isoforms revealed striking conservation of the DSX DM domain and the female-specific carboxy domain encoded by the 2.0 kb product. Through 5'-3' RACE PCR, we next obtained longer cDNAs containing fully open reading frames. The 3 cDNA clones were named as *Aeadsx*^*F1 *^(2846 bp), *Aeadsx*^*F2 *^(2384 bp) and *Aeadsx*^*M1 *^(1918 bp), which encoded three *Aedes aegypti *DSX isoforms, DSX^F1 ^(278 aa), DSX^F2 ^(267 aa) and DSX^M1 ^(548 aa), respectively. The alignment of the *Aeadsx *cDNA sequences with the *Aedes *corresponding genomic sequence was used to define the exon/intron organization and the alternative splicing events leading to distinct sex-specific mRNAs (Figure 1A).

These data confirmed the identification of the *Ae. aegypti dsx *gene, which produced sex-specific transcripts by alternative splicing.

### *Aeadsx *genomic organization and evolution

The *Aeadsx *gene spans a very large 450 kb long genomic region, located in supercontig 1.370, and consists of at least eight exons with seven introns that vary markedly in length from 208 to 274,879 bp.

The first four *Aeadsx *exons (2-3a-3b-4) are non-sex-specific and encode the 248 amino acid common N-terminus region of AeaDSX proteins. The four exons are followed by two alternatively spliced female-specific exons (5a-5b), encoding 30 and 19 amino acid sequences, respectively, and two male-specific exons (6-7), the first one encoding the male-specific protein domain of 300 aa and the second one constituting the 3' UTR sequence (Figure 1A).

**Figure 2:**
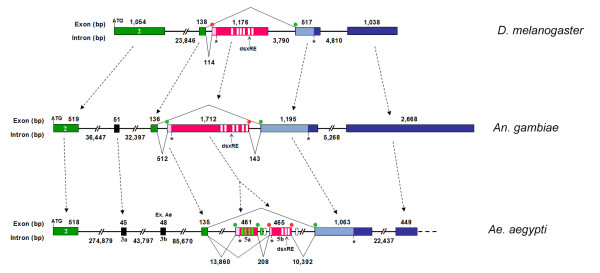
**Comparative genomic structure of the *D. melanogaster*, *Ae. aegypti *and *An. gambiae dsx *genes**. Comparative genomic structure of the *D. melanogaster*, *Ae. aegypti *and *An. gambiae dsx *genes. Green boxes represent the OD1 and OD2 domain-encoding exons. Black boxes represent exons encoding protein regions conserved in mosquitoes but not in fruit flies. Alternative male-specific and female-specific exons are represented as blue boxes and pink boxes, respectively. Green dots represent canonical acceptor/donor splicing sites. Red dots represent weak acceptor/donor splicing sites. White and green rectangles represent, respectively, TRA/TRA-2 binding sites and *Nasonia *dsxRE. In *Drosophila*, the *Dmdsx *gene is located in a 45-kb region on chromosome 3R and is organized into six exons and five introns, with three common exons followed by a female-specific and two male-specific exons. *Dmdsx*^*F *^translation initiates at the AUG within exon 2 and terminates within the female-specific exon 4, while in the case of *Dmdsx*^*M*^, translation begins at the same AUG and terminates within the first male-specific exon 5.

**Figure 3:**
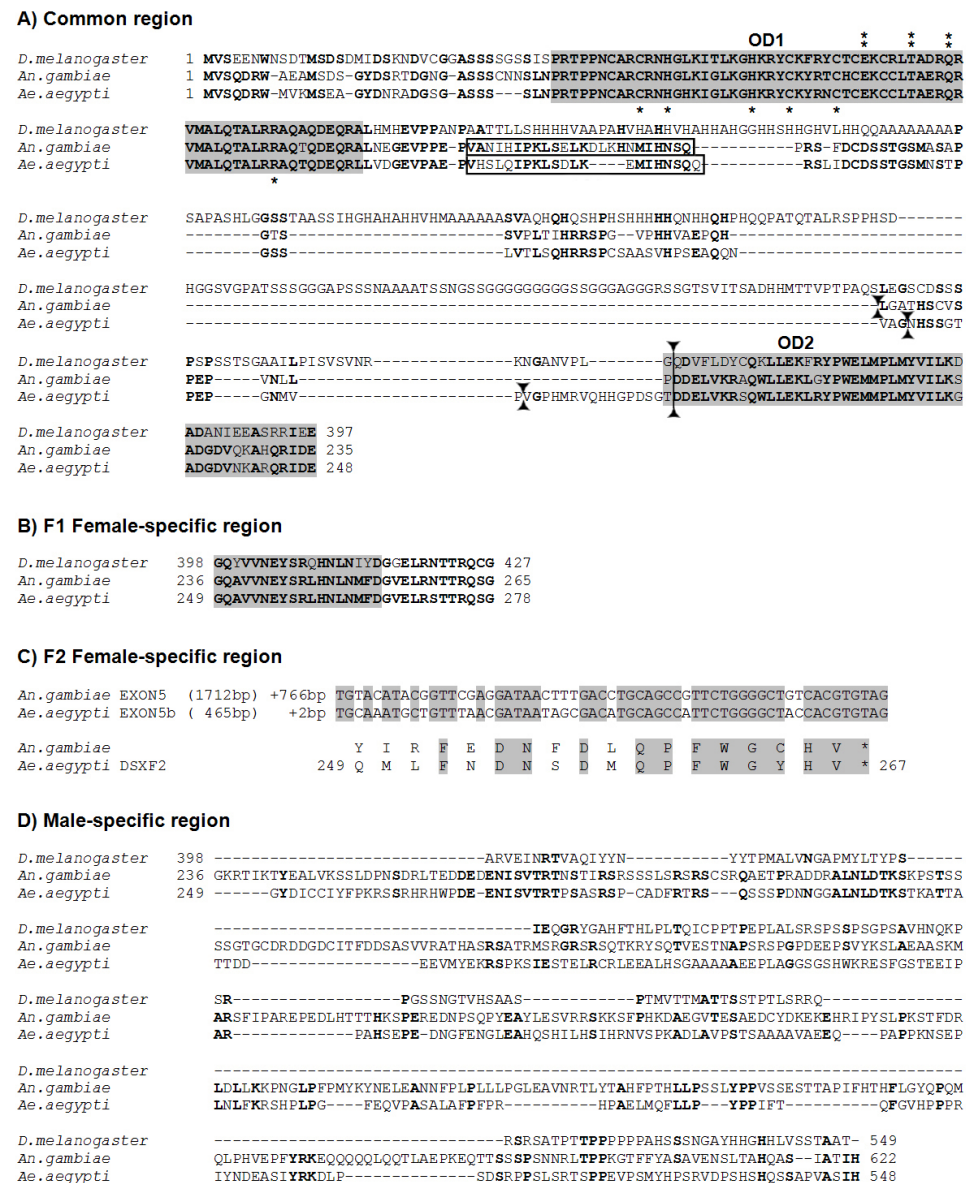
**Multiple sequence alignment of DSX homologues**. Protein sequence alignment of DSX isoforms in *Drosophila melanogaster*, *Anopheles gambiae *and *Aedes aegypti*. The sequences are divided into a region that is common to males and females (A), a first female-specific region (B), a second female-specific region (C) and a male-specific region (D). The amino-terminal DNA binding (OD1) and oligomerization domains (OD2) are boxed in grey. The asterisk (*) indicates six amino acids whose replacements has been shown to abolish DNA-binding activity in *D. melanogaster*; (**) double asterisks indicate the three amino acids specific for the DSX DM domain. Intron positions are indicated by solid triangles. The amino acid stretch marked in rectangular box corresponds to the 63-bp sequence removed in some but not all *Aeadsx *transcripts. This event leads to the in-frame deletion of the indicated 21-amino acid tract (see Figure 5B.3 for further details). Also the conserved removed amino acid stretch of *An. gambiae *is marked in rectangular box. Bold letters indicate amino acid identity in the homologous proteins. Gaps were introduced in the alignments to maximize similarity. The comparison of protein sequences was performed using Clustal-W (1.82).

The first four *Aeadsx *exons correspond by homology to the second, third and fourth exons of the *Angdsx*, suggesting that we missed the real first *Aeadsx *exon (Figure 2). Interestingly, some of the *Aedes dsx *cDNAs exhibit in this second exon the presence of a non-sex-specific alternative splicing event by intron retention of a short coding 63-nt region. The corresponding encoded 21-amino acid tract is highly conserved in AngDSX isoforms (Figure 1 and 3A) but lacks any conservation with DSX isoforms from other dipteran species (data not shown). This finding suggests that this short region is used *in vivo *and should play a functional role that seems to be under positive natural selection.

Because the N-termini of the encoded *Anopheles *and *Aedes *DSX proteins are highly conserved, starting from the first putative methionine, this selective constraint suggests that the *dsx *translation start site is conserved in both species. As in the 5' UTR of the isolated cDNA there is lack of sequence corresponding to the *Angdsx *exon 1, and additional upstream genomic sequences of *Aeadsx *are likely still to be identified, including the promoter region.

The *Aedes *exon 3a and 3b exhibit a low level of amino acid conservation. In particular, exon 3a seems to correspond to the unique *Anopheles *exon 3 (see alignment in Figure 3). The short exon 3b encodes 16 amino acids with no homology to DSX or other known proteins and it constitute an event of exon gain. Exon 5 in *Aedes *is separated into two exons (5a and 5b). These exons (461 bp and 465 bp, respectively) maintain the female-specific alternative splicing regulation (Figure 2). Interestingly, it seems that *dsx *underwent to either intron gain in the *Aedes *or intron loss in the *Anopheles *lineages.

Finally, *Aedes *exons 6 and 7 (1.1 kb and 0.5 kb) correspond to the *Anopheles *male-specific exons 6 and 7. The exon 6 encodes in both species the male-specific DSX portion and includes part of the 3' UTR. The exon 7 corresponds to the remaining 3' UTR.

The *Aedes dsx *exons show variable sequence similarity, conserved exon-intron junctions, an exon gain (3b exon) and an intron gain (intron between 5a and 5b exons) events and hence partial structural correspondence to the *Anopheles dsx *exons. In particular, with respect to the *Anopheles *homologue, *Aeadsx *common exons 2 and 4 are largely conserved in size and content, while sex-specifically regulated exons as well as exons 3a and 3b share a lower level of sequence identity. On the contrary, an extensive divergence in intron structure (intron position and length) was observed that reflects overall genomic differences between the species (Figure 2). The *dsx *gene in *An. gambiae *is contained within an 85-kb genomic region; therefore, the corresponding *Ae. aegypti *genomic sequence is approximately 5.3-fold larger. This difference is due to the presence of very large introns in the *Ae. aegypti *homologue, with an average intron size of 64 kb in contrast to the observed average intron size for *Angdsx *(15 kb). This is undoubtedly reflective of the overall differences in genome organization of the two species because the *An. gambiae *genome size is about 243 Mb, while *Ae. aegypti *is about five-fold larger at about 1.31 Gb. Most of this difference is due to the high frequency of repetitive sequences in the *Ae. aegypti *genome [26].

An analysis of *Aeadsx *introns with CENSOR software (http://www.girinst.org/censor/index.php) [30] revealed the presence of multiple copies of repetitive elements, the most abundant of which are the NON-LTR/Jockey LINE-1_AA elements [31], detected in 34 copies, and the NON-LTR/SINE Feilai element [32], detected in 30 copies (Additional file 1, Table S1). Interestingly, the comparative analysis of CENSOR outputs of *Aeadsx *introns revealed that two out of three sex-specifically regulated introns, the short intron 5 (208 bp long) and intron 6 (10392 bp long), significantly deviate in the number of repetitive elements per kb (indicated as NoRE/kb) and in the percentage of nucleotides of repetitive elements relative to intron nucleotides (indicated as REbp). *Aeadsx *intron 5 contains no repetitive element at all, while *Aeadsx *intron 6 presents a NoRE/kb value of 0.6 and a REbp value of 7% with respect to the mean NoRE/kb and REbp values of non-sex-specific introns, which were 1.13 and 18%, respectively (Additional file 2, Figure S1). This finding suggests that there may have been positive selective pressure on these two intronic regions against repetitive elements to preserve sex-specific alternative splicing regulation.

We used the entire supercontig 1.370 *dsx*-containing region of *Ae. aegypti *to analyze the presence and nature of genomic microsynteny between *Ae. aegypti *and *An. gambiae*. We compared the amino acid sequences of all putative genes in *Aedes *supercontig 1.370 with putative genes of the syntenic region in *An. gambiae *obtained from the Ensembl precomputed tBLAT DNA-DNA comparison for the two insect species.

Gene density in these regions was apparently higher (twice) in *An. gambiae *relative to *Ae. aegypti*. The relative synteny quality, expressed as a percentage and calculated by dividing the number of conserved genes in both syntenic regions by the total number of genes in both regions [33], was 62%. Out of a total of 16 genes in these regions, 10 homologues were found including *dsx*. Interestingly, microsynteny of the *prospero *gene with the *dsx *gene, previously described also for *Anopheles gambiae*, *Apis mellifera *and *Tribolium castaneum *[23], was not conserved in the *Aedes aegypti dsx*-containing region (Additional file 3, Figure S2). This finding suggests that a chromosomal rearrangement may have occurred after the split between the two species, bringing the *Aedes prospero *gene into a different genomic position outside of supercontig 1.370 that is not yet mapped on any *Aedes *chromosome. A putative *Aedes prospero *gene (AAEL002769) is located in supercontig 1.67, and a mapped chromosome position is not yet available for this supercontig.

In *Aedes aegypti*, a putative gene (AAEL009111) encoding a phosphodiesterase is located in *Aeadsx *intron 2 (position 918233-980385 of supercontig 1.370) in the opposite direction of transcription with respect to *Aeadsx*. The *Anopheles *homologue of this gene (AGAP004054) is located downstream of the *prospero *gene in the *Angdsx*-containing microsyntenic region, in the opposite direction of transcription with respect to the *Aedes *counterpart. In this case a rearrangement, occurred during evolution, moved the gene from one position nearby the *dsx *gene into the gene itself or vice versa.

### Comparison of AeaDSX isoforms

The alignment of AeaDSX isoforms with the DSX isoforms of *D. melanogaster *and *An. gambiae *is presented in Figure 3. *Drosophila *DSX proteins essentially contain two domains, OD1 and OD2, which serve as interfaces for protein and DNA interactions [4,34]. The non-sex-specific OD1 is composed of an atypical zinc-finger domain (DM), which directly binds to target sequences on the DNA. OD2 is an oligomerization domain that has a common region and a female-specific portion. A sequence alignment of *Aeadsx *isoforms shows a high degree of sequence conservation in the N-terminus up to the unique OD1 domain and the common part of the OD2 domain. Furthermore, within the OD1 domain, we have found full conservation of the six residues (C, H, H, C, C, R) essential for DNA-binding activity in *D. melanogaster *[35] and the three residues (E, T, Q) recently identified to be specific to the insect DSX DM domain [23]. These nine residues were also conserved in *An. gambiae *DSXs [19].

The region that links OD1 and OD2 is less conserved in *Aedes *and *Anopheles *and lacks the low complexity region from *Drosophila *that contains a large number of histidine, glycine and alanine residues (Figure 3A).

The C-terminal region of DSX^F ^proteins shows a very high degree of sequence conservation among different insects, and this region in *Aedes aegypti*, encoded by the *Aeadsx*^*F1 *^transcript, is also very highly conserved (Figure 3B).

The predicted protein encoded by the *Aeadsx*^*F2 *^transcript differs from the AeaDSX^F1 ^protein due to a short 19-aa alternative female-specific C-terminus with no obvious similarity to other DSX isoforms. However, the corresponding nucleotide sequence encoding the short AeaDSX^F2 ^C-terminal region is highly conserved in the untranslated region of the *Anopheles gambiae *female-specific *dsx *exon 5 (Figure 3C). This strong sequence conservation of an untranslated region of the *An. gambiae dsx *gene could be related to either the use of this region for alternative splicing events (still to be identified in *Anopheles*) leading to a coding frame in *Anopheles*, as in *Aedes*, or to the involvement of this region in the splicing mechanism underlying sex-specific regulation of this region (see below).

The predicted male-specific protein encoded by Aeadsx^M1 ^is remarkably poorly conserved, displaying only very short stretches of similarity, as observed for *Drosophila *(Figure 3D) and others dipteran species (data not shown).

Finally, paralog search in the *Aedes aegypti *genome via the BLASTP matching function, using the three AeaDSX isoforms as virtual probes, revealed a single putative paralog gene (AAEL004696). However, this gene encodes a protein which shares with AeaDSX isoforms only a very well-conserved DM domain. This DM domain exhibits conservation of only one out of three DSX-DM domain-specific amino acids identified by Oliveira *et al.*, [23].

### Phylogenetic relationship and molecular evolution of the *Aedes aegypti dsx *gene

As remarked above, the dipteran DSX protein is essentially characterized by the two OD1 and OD2 domains, which constitute the most conserved portion of the *dsx *gene across species. The high degree of conservation of these two domains is expected according with their regulative role in protein-protein and protein-DNA interactions.

**Figure 4:**
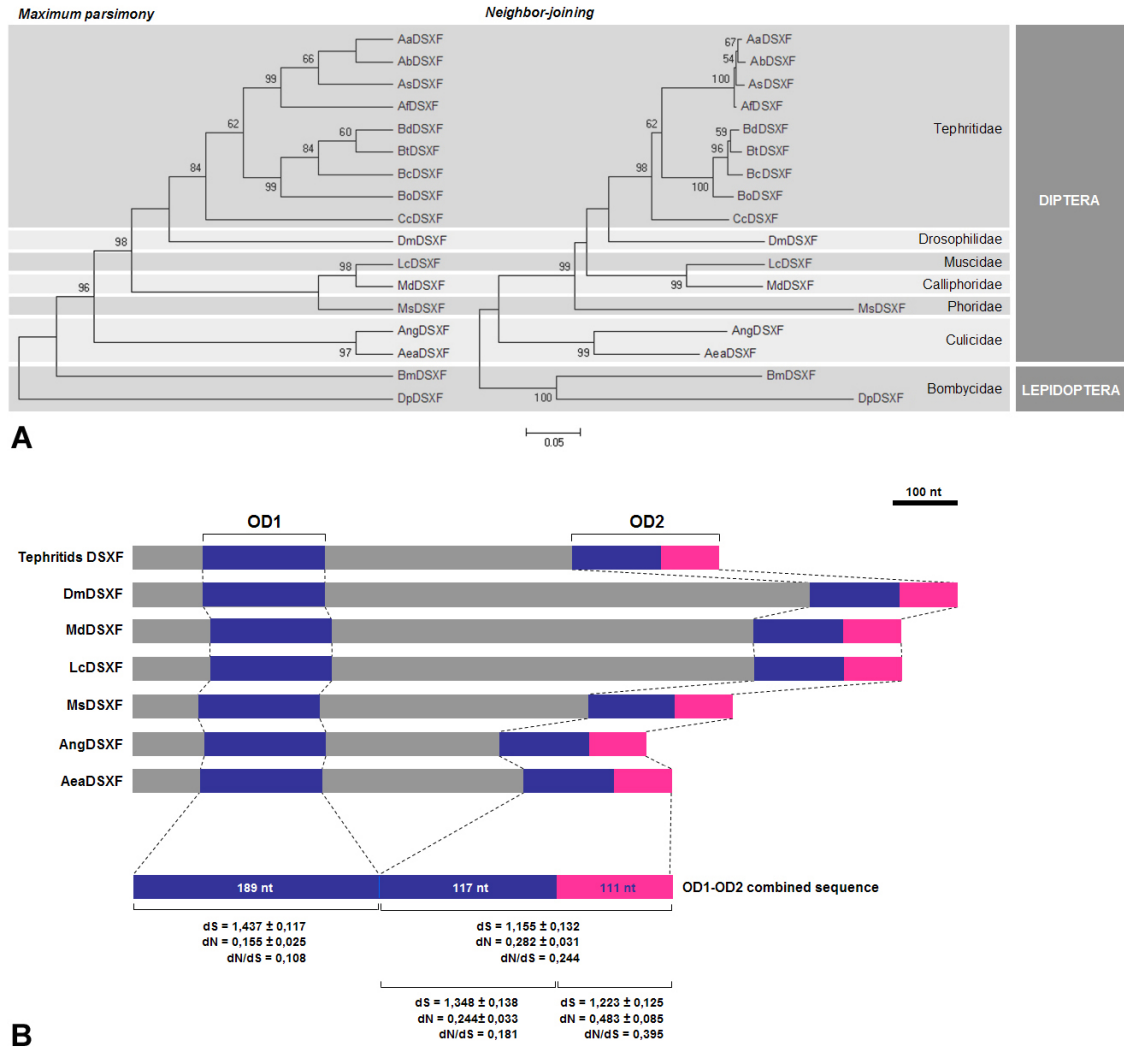
**Phylogenetic and molecular evolutionary analyses**. (A) A phylogenetic tree based on the combined *dsx *nucleotide sequences encoding OD1 and OD2 domains in six dipteran families and two lepidopteran species. The consensus of six equally parsimonious trees (tree length = 757 and parsimony-informative characters = 206) and the neighbor-joining tree (417 total characters) obtained using the combined nucleotide sequences are shown with bootstrap support above branches (shown only when greater than 50%). Taxonomic relationships are indicated in the right margin of the trees. The topology was rooted with the *dsx *corresponding sequences from the lepidopteran *B. mori *and *Danaus plexippus*. (B) Comparison of dipteran female-specific *dsx *coding sequences and localization of OD1 and OD2 domains. Pairwise synonymous (dS) and non-synonymous (dN) substitution rates and the mean pairwise ratio (dN/dS) values are placed above the corresponding coding sequence.

To determine the phylogenetic position of AeaDSX^F1^, we defined a combined *Aedes aegypti dsx *nucleotide sequence, containing regions encoding respectively the non-sex-specific OD1 domain and the complete OD2 domain (non-sex-specific portion and the female-specific portion; nucleotides 94 to 288 joined with nucleotides 607 to 837 of GenBank DQ440532 CDS), and we aligned it with the corresponding homologous regions of *dsx *sequences from fourteen other dipteran species: *Drosophila melanogaster*, *Ceratitis capitata*, *Bactrocera tryoni*, *B. correcta*, *B. dorsalis*, *B. oleae*, *Anastrepha bistrigata*, *A. serpentina*, *A. amita*, *A. fraterculus*, *Anopheles gambiae*, *Musca domestica*, *Lucilia cuprina *and *Megaselia scalaris*. Figure 4A shows the maximum parsimony and neighbor-joining trees for this alignment. We used the lepidopteran *Bombyx mori *and *Danaus plexippus dsx *sequences to root both trees. The resulting topologies agreed well with the taxonomy of the order Diptera and showed high confidence levels in the groups defined. As expected, based on phylogenetic relationships, both dendrograms for the combined *dsx *nucleotide sequence grouped *Aedes aegypti *with *Anopheles gambiae*, and all the remaining dipteran species formed another group, while the lepidopteran *Bombyx *and *Danaus *representatives clustered in a basal clade.

Our analysis, performed with combined OD1/OD2 sequences, confirms the topologies obtained for insects *dsx *genes by using the whole DSX^F ^or DSX common portions encoding sequence [36,37] the combined OD1/non-sex-specific OD2 portion sequences [23].

We utilized the same dipteran *dsx *OD1-OD2 combined nucleotide sequences to perform an analysis of the nucleotide variation across corresponding coding regions to examine whether the sequences evolve under purifying constraint or positive selection for amino acid changes. These analyses were conducted on the OD1 and OD2 coding sequences separately and on the OD2 coding sequence partitioned into the common region and the female-specific region (Figure 4B).

Even though diffuse purifying selection was detected in both OD1 and OD2 domains, the OD2 domain showed a relaxation of selective constraints compared to the OD1 domain. The difference between the dN/dS values was due to the non-synonymous substitution rate, in particular in the female-specific region where dN was significantly higher than the dN values of the OD1 domain and the common part of OD2 domain. These findings suggest that OD1-OD2 domains of the DSX^F ^protein have stringent structure/function relationships leading to a constrained evolution. However, the female-specific region of the OD2 domain exhibits a lower level of constraint. Amino acid changes in the female-specific portion of the DSX protein that affect dimerization dynamics might induce changes in the transcriptional regulation of *dsx *target genes (possibly including heterochronic, heterotopic, heterometric or heterotypic variations, as well as recruitment/loss of specific target genes).

Hence, it is conceivable that even though DSX belongs to a highly conserved transcription factor protein family, it could be involved in the adaptive process and respond to the key force in insect evolution, sexual selection, by changing those protein regions involved in the control of expression of genes affecting dimorphic phenotypic traits in the two sexes.

### Developmental expression analysis of *Aeadsx*

**Figure 5:**
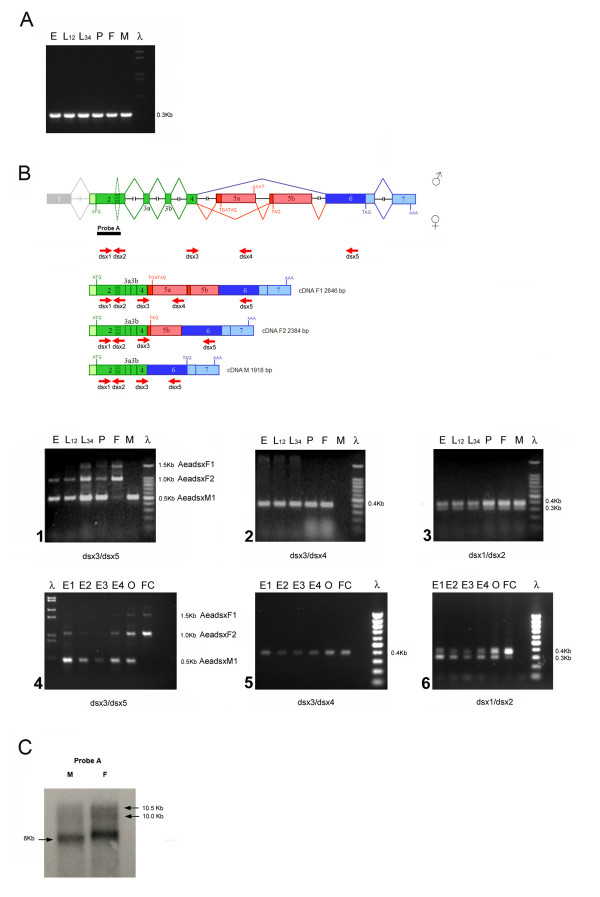
**Developmental expression analyses of the *Aeadsx *gene**. The analyses were performed on the following samples: E1 = 0-1.5 h embryos; E2 = 1.5-2 h embryos; E3 = 2-5 h embryos; E4 = 8-12 h embryos; E: 0-36 h embryos; O = dissected ovaries; FC = female carcasses depleted of ovaries; L^12^= early larvae; L^34^= late larvae; P = pupae; M = adult males; F = adult female. Except for M, F, O and FC all samples are composed of mixed sexes. Negative controls are not shown. (A) Amplification of *Ae. aegypti rp49 *transcripts with the Aearp49+/Aearp49- primer pair. The *Aedes aegypti *ribosomal gene *rp49 *is constitutively expressed throughout development. (B) *Aeadsx *developmental expression pattern. (B.1 and B.4) The dsx3/dsx5 primer combination amplified at adult stages a 0.5-kb male-specific cDNA fragment and two female-specific cDNA fragments (1.0 kb and 1.5 kb). These three bands were detected in pupae and late larvae, while the 1.5-kb band was absent in embryos and mid-larvae but present in ovaries and female carcasses. (B.2 and B.5) The dsx3-dsx4 primer combination amplified at adult stages a female-specific cDNA fragment. A cDNA product of identical size was amplified at all developmental stages, including embryos, suggesting an early *Aeadsx *female-specific regulation. (B.3 and B.6) The dsx1/dsx2 primer combination amplified in all samples two slightly different cDNA fragments (0.37 kb and 0.31 kb), corresponding to the alternatively spliced isoforms of exon 2 either containing (0.37 kb) or not containing (0.31 kb) the 63-bp intronic sequence. In contrast to the data reported in Figure B.1-3, the RT-PCR results in Figure B.4, B.5 and B.6 lack of a positive semiquantitative control and the apparent changes in expression levels of *Aeadsx *isoforms during embryonic stages have to be further investigated. (C) A northern blot analysis was performed on total RNA (20 μg) extracted from male and female *Ae. aegypti *adults. The genomic position of the utilized probe is indicated in Figure 5B. The observed molecular size of *Aeadsx *transcripts confirms that isolated *Aeadsx *cDNA clones were not full-length at the 3' and 5' ends.

To analyze the developmental expression pattern of the *Ae. aegypti dsx *gene, an RT-PCR analysis was performed on total RNA extracted from different stages of development using primer pairs spanning the *Aeadsx *sex-specifically regulated region. The recently isolated *Aedes aegypti *(*Aearp49*) homologue of the *Drosophila rp49 *constitutively expressed gene was tested as the positive control for the RT-PCR analysis in non-saturating conditions [38-42]. *Aearp49 *was constitutively expressed from the embryonic stage of *Aedes *until adulthood, as in the fruitfly (Figure 5A).

In *Drosophila, dsx*^*M *^mRNAs are not readily detected in 3-10-hour-old embryos of either sex but are easily detected in 10-16-hour and 16-22-hour-old male XY embryos [43]. These authors showed that in male embryos, subsets of male somatic gonad cells were clearly stained with anti-DSX^M ^during embryonic stage 13. In *Anopheles gambiae*, sex-specifically spliced *dsx *mRNAs are detected in sexed adult mosquitoes, but no early developmental stages were investigated [19].

RT-PCR of *Aedes *with dsx3/dsx5 primers amplified 0.5-kb and 1.0-kb cDNA products from very early embryonic stages (see Figure 5B.4) and an additional 1.5-kb cDNA product from late larvae (3^rd^-4^th ^instars). During pupal stages, the 1.5-kb cDNA product was highly reduced, as well as the 1.0-kb cDNA product (Figure 5B.1 and Figure 5A as control). At adult stages in sexed mosquitoes, the longer 1.0- and 1.5-kb cDNAs were detected only in females, while the 0.5-kb cDNA was detected only in males. Sequence analysis of the cDNA products showed that they were *Aeadsx *alternatively spliced transcripts encoding three different protein isoforms and corresponding in structure to those shown in Figure 1A. Sequence analysis of these cDNA products showed that exons 5a, 5b and 6 are included in the same mRNA, which is detected in late larvae and at a lower level in pupae and in adult females. Transcripts including exons 5b and 6, but not exon 5a, are detected from early embryonic stages during all development and in adult females. Transcripts including exon 6 (encoding the male-specific DSX C-terminus) but not exons 5a and 5b are detected from early embryonic stages throughout development and in adult males. Hence at least at adult stages, both 5a and 5b exons are female specific, while exons 2, 3a, 3b, 4 and 6 are not sex-specific. Furthermore, it seems that exon 5a is used only from late larval stages, while exon 5b is present already in early *dsx *mRNA.

Although it is not presently possible to sex *Aedes *embryos or early larvae, the length correspondence of the adult sex-specific cDNA products with the respect to those detected at early stages of development suggests that the early sex-specific *dsx *expression observed in *Drosophila *is conserved not only in the other dipteran species *Ceratitis capitata *[44] but also in the more distantly related *Aedes aegypti*.

In dissected ovaries, the dsx3/dsx5 primer pairs detected the three *Aeadsx *cDNA fragments amplified from late larval stages (Figure 5B.4). The shorter 0.5-kb cDNA product, which corresponded in adults to a male-specific mRNA, was surprisingly present in a female-specific tissue, namely the ovaries. However, this cDNA product was absent in RT-PCR performed on the ovary-depleted female somatic carcasses (Figure 5B.4). The dissected females were young but not all were virgins. Furthermore, during the dissection of the ovaries, portions of the oviducts and uteri could have been included, leading to the possible presence of fertilized eggs in the dissected material. Because the dsx3/dsx5 primer pair also detected the same 0.5-kb cDNA product in 1.5 h embryos, it is possible that this product was absent in the ovaries but present in the fertilized eggs and hence not excluded in the final dissected samples.

**Figure 6:**
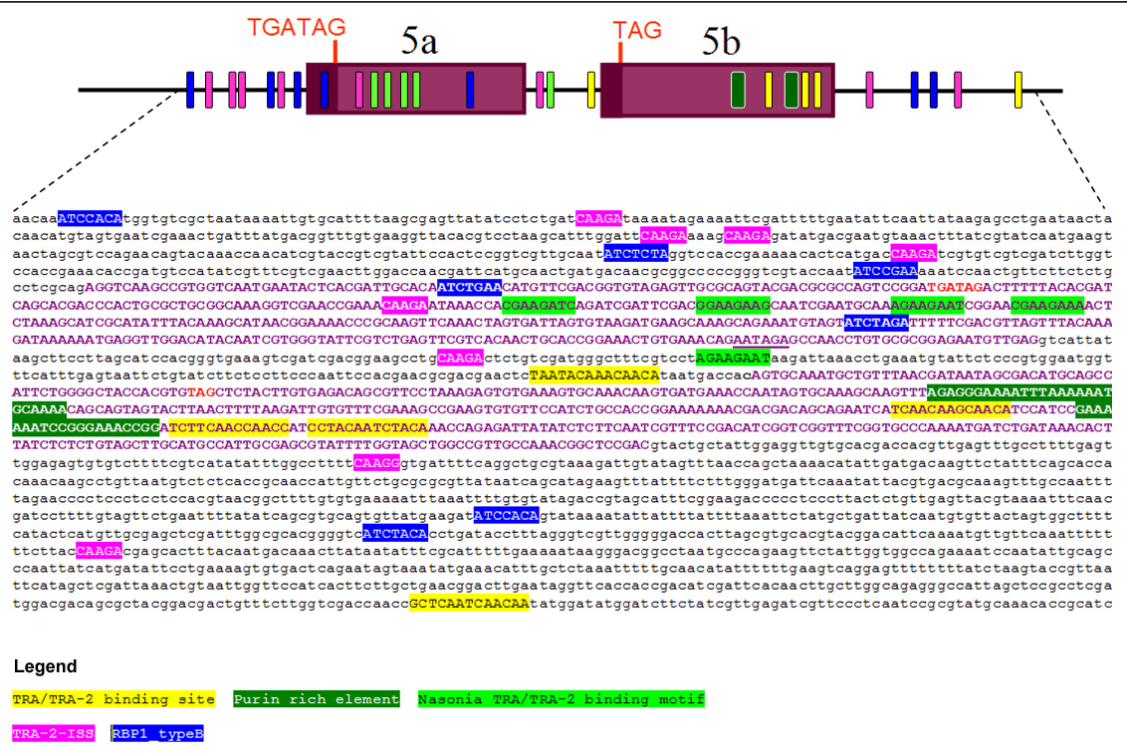
**Putative *cis*-acting elements of the *Aeadsx *gene**. The distribution of the putative *cis*-acting elements in the sex-specifically spliced region of *Aeadsx *is reported. Black lowercase letters indicate intronic regions. Dark purple letters indicate the female-specific 5a and 5b exons. Translational stop codons are reported in red uppercase letters. The putative polyadenylation signal of exon 5a is underlined.

We then used two other primer pairs to extend the RT-PCR expression analysis. The dsx3/dsx4 pair led to amplification of the expected female-specific 0.4-kb long cDNA product in adult females (see Figure 5B.2). This cDNA product was amplified at larval and pupal stages, confirming the previous data that showed the presence of exon 5a during this developmental period. However, the 0.4-kb cDNA fragment was also amplified at embryonic and 1^st^/2^nd ^instar larval stages, indicating that exon 5a is present in mRNAs earlier than previously shown (see Figure 5B.2 and 5B.5). A possible explanation of this apparently contrasting data is that there is a third alternative mRNA in which exon 5a is present but exon 5b and exon 6 are not. This mRNA could be generated by alternative polyadenylation at the 3' UTR of exon 5a. A northern blot analysis of total RNA from sexed adult mosquitoes showed the presence of a third female-specific *Aeadsx *transcript, offering support to this explanation (Figure 5C). The two mRNAs including exon 5a are different in the 3' UTR but potentially encode the same DSX^F1 ^protein. Furthermore, a putative polyadenylation signal (AATAGA) was identified *in silico *333 bp downstream of the stop codon present in exon 5a (Figure 6). Interestingly, the *Apis mellifera dsx *homologue produces two female-specific transcripts using two alternative polyadenylation signals, and possibly as in *Aedes*, they both encode the same DSX^F ^protein [21].

dsx1/dsx2 primers amplified in all samples two slightly different cDNA fragments (0.37 kb and 0.31 kb) corresponding to the alternatively spliced isoforms of exon 2 either containing or not containing the 63-bp intronic sequence (see Figures. 3A, 5B.3 and 5B.6). Interestingly, the relative ratio of the two RT-PCR products is dynamic during development, with the longer one becoming more prominent from pupal until adult stages (Figure 5B3 and Figure 5A as control). These data suggest that this *Aeadsx *intron-retention splicing event is subjected to stage-specific regulation. It would be of interest to check in future if also in *Anopheles *during development there is a similar *dsx *expression pattern, concerning this small exon/intron.

Sex-specific expression of the *Aeadsx *gene was further confirmed by northern blot hybridizations. A probe of 500 bp corresponding to the non-sex-specific region (Probe A) was hybridized on a northern blot of total RNA extracted from sexed adults. The probe identified three female-specific transcripts of 10.5 kb, 10.0 kb (weaker signals) and 8.5 kb (prominent signal) and an 8.0-kb male-specific transcript (Figure 5C).

### Putative splicing regulatory elements of *Aeadsx *gene

In *D. melanogaster*, the female-specific splicing of *dsx *pre-mRNA requires the binding of the splicing regulators TRA (which is female-specifically expressed) and TRA-2 (an auxiliary factor expressed in both sex) to six repeats of the dsxRE element and to one PRE (purine rich element) element, which is present only in the untranslated region of the *dsx *female-specific exon (Figure 2). TRA and TRA-2 activate a weak (due to the presence of purine nucleotides in its polypyrimidine tract) 3' splicing acceptor site that precedes the female-specific exon [45,46]. The activity of TRA/TRA-2 is not required for the processing of pre-mRNA in males, which constitutes the default type of splicing. This *dsx *splicing regulation and the dsxRE element (13 nt long) are highly and widely conserved in many dipteran species (for review, see [3]).

Novel types of regulatory *cis*-elements have recently been identified in *dsx *homologues of three non-dipteran species, such as the 20-bp CE1 element in the lepidopteran *Bombyx mori dsx *gene [47] and the 8-bp repeat in the *tra/fem*-regulated *dsx *of *Nasonia vitripennis *and *Apis mellifera *[48]. These results revealed the potential evolvability of *dsx *splicing paralleled by changes in the regulatory elements and the corresponding upstream splicing regulators. The CE1 element is indeed recognized by the PSI protein, which negatively regulates female-specific splicing in males [47]. The conserved 8-nt *Nasonia*/*Apis *repeat element [(T/G)GAAGAT(T/A)] was suggested by Verhulst *et al. *(2010) to be a potential TRA/TRA-2 binding motif. However, there is no sequence similarity between this hymenopteran sequence and the 13-nt *Drosophila *TRA/TRA-2 binding element, known also as dsxRE [TC(T/A)(T/A)CAATCAACA], which is on the contrary conserved in all investigated dipteran species. This hymenopteran type of repeat element, which, in two distantly related hymenopteran species, appears to be conserved in sequence and clustered distribution, is likely a novel key regulatory element involved in the sex-specific splicing regulation of *dsx*. To avoid confusion, we referred to this novel element as *Nvdsx*RE (*Nasonia vitripennis dsx *repeat element). NvdsxRE is also present in *Nasonia tra *[49] and *fru *[48], as well as, in *Apis mellifera fem *(not shown) *dsx *and *fru *[48,50], strongly suggesting its involvement in sex-specific alternative splicing regulation. Hence, an upstream common splicing factor is expected to be present in hymenopterans, simultaneously controlling all three downstream target genes (*fem*/*tra*, *dsx *and *fru)*.

An *in silico *analysis of the intronic donor/acceptor splicing sequences of the *Aedes dsx *gene was performed (Table 1) and revealed that almost all sequences have the conserved GT-AG motifs. Interestingly, in the splice-acceptor site preceding the female-specific exon 5b, the AG was substituted by GT, strongly indicating a significant deviation and hence a potentially regulated 3' splicing acceptor site. We thus defined more precisely the general consensus of the 3' acceptor splicing sites in the *Aedes aegypti *genome through the tabulation of 4688 random intron sequences extracted from the Ensembl database using the BIOMART tool. We derived a consensus number of 8.02 ± 2.15, a value equivalent to the one observed in the *Anopheles *genome based on 215 introns [19] (see Table 1). In the *Aeadsx *gene, pyrimidine stretches in most of the 3' splice sites do not deviate significantly from this consensus. However, the pyrimidine stretch preceding the female-specific exon 5b deviates significantly from the consensus (5/12), suggesting that this sequence is a suboptimal 3' splicing acceptor site.

**Table 1:** Exon-intron junctions of the *Aeadsx *gene

*Aeadsx gene*					
**Exon No.**	**Exon size(bp)**	**Splice-donor**	**Intron size(bp)**	**Splice-acceptor**	**N° of Y**
1?	---	---	---	---	
					
2	518	A G	274879	N H	12
		TTGCAG/gtaggtgtgaggcata		tctcctctcttttcag/GCAACC	
3a	45	P V	43797	G P	10
		TACCAG/gtgagttcgctgttga		tctcttctggtttcag/TTGGCC	
3b	48	T D	85670	D E	9
		GAACAG/gtgcgtacttccttaa		tcgtttccaatttcag/ACGACG	
4	135	E G	13860	Q A	11
		ACGAAG/gtatgggggttcttac		cttctctgcctcgcag/GTCAAG	
5a	461	n.c.	208	Q M	5
		gttgag/gtcattataagcttcc		acataatgaccacagt/GCAAAT	
5b	465	n.c.	10392	Y D	8
		cgacag/gtactgctattggagg		tcaatccctcaaacag/GATACG	
6	1063	n.c.	22437	n.c.	11
		acgaag/gtgagtgttctttttt		ctcttttcttcaacag/tttcac	
7	449	n.c.	---	---	---
		tacacacgtacacattaaaatag			
					
Consensus		AG/gtRagt		YYYYYYYYYYYYNYag/G	8,02 +/- 2,15
		C			

The exon/intron/exon boundaries of *Aeadsx *gene are reported. Exonic coding sequences are shown in uppercase letters, and non-coding regions (n.c.) are in lowercase letters. Encoded aminoacids are reported above the first base of each triplet. R = A or G nucleotide. Y = T or C or nucleotide. N = any nucleotide. The number of pyrimidines (Y) in the 12 bp preceding the 3' splice-acceptor site (NYag) is indicated. The consensus number of pyrimidines (8.02) was derived from the analysis of 4688 *Ae. aegypti *splice-acceptor sites. The *Aeadsx *gene intron 5 (13,860 bp) represents the intron preceding the female-specific exon 5a, and its polypyrimidine stretch does not deviate significantly from the consensus. The splice-acceptor site of the short 208-bp intron preceding the female-specific exon 5b is a weak acceptor site, as is the female-specific exon 5b splice-donor site, which deviates significantly from the consensus sequence.

Analysis of the 5' splice donor sites of *Aeadsx *introns revealed that most of the sites are conserved in their consensus sequence (GTRAGY - Stephens *et al. *1992). Indeed, the 5' splice donor site at the end of the female-specific exon 5b presents only three conserved positions out of six [51], suggesting again that this site could constitute a weak splice donor site requiring positive splicing regulation by an enhancer.

We then analyzed the *Aeadsx *genomic sequence, including the two female-specific exons 5a and 5b and the surrounding intron regions, using the MACAW alignment software [52] to identify putative *Drosophila*, lepidopteran and hymenopteran *cis*-acting elements potentially involved in splicing regulation of *Aeadsx*. We utilized the *Drosophila dsx *TRA/TRA-2 binding site and PRE element, the Bombyx CE1 element and *Nvdsx*RE element as virtual probes. We found the following:

1) Five copies of TRA/TRA-2 binding site sequences exclusively in the female-specific exon 5b. Interestingly, of the five repeats, three were located only 140 bp and 1 repeat 1 kb away from the potentially regulated weak 5' splice donor site at the end of the female-specific exon 5b (Figure 6). The fourth repeat was within the short intron 6 (208 bp), which separates the two female-specific exons, and the fifth repeat was located 1 kb downstream of the female-specific exon 5b in intron 7 (Figure 6). This is a strong indication of their potential involvement in the female-specific splicing of this *Aeadsx *region. The sequences of the five putative *Aedes *TRA/TRA-2-binding sites are more similar to the *Drosophila *consensus than to the TRA/TRA-2 binding sites found in the *Anopheles dsx *homologue. The deviations in the sequences occur within the first four bases of the *Aedes *TRA/TRA-2-binding sites (See Additional file 4 - Table S2).

2) Two putative PRE elements exclusively in the female-specific exon 5b.

3) A cluster of four conserved *Nvdsx*REs exclusively in the female-specific exon 5a of the *Aeadsx *gene. The sequence conservation in an untranslated region and its clustering within the regulated genomic region strongly suggest a functional relevance of this element. A further copy of the *Nvdsx*RE repeat is located in the 208-bp intron between *Aeadsx *female-specific exons 5a and 5b. A similar search for *Nvdsx*REs was also performed for the *Anopheles *and *Drosophila dsx *and *fru *homologues but failed to identify any putative conserved element.

We extended our analysis to look for the presence of two more *cis*-acting elements in the *Aeadsx *gene, the RBP1 binding site and the TRA-2-ISS element. In *Drosophila*, RBP1, a non-sex-specific SR splicing factor, contributes to the activation of female-specific *dsx *splicing *in vivo *by recognizing two RBP1 target sequences (Type A: DCADCTTTA; Type B: ATCYNNA, [53]) within the purine-rich polypyrimidine tract of the weak female-specific 3' splice site [54]. Furthermore, the expression of functional TRA-2 protein in the male germline of *Drosophila *is regulated through a negative feedback mechanism, which is evolutionarily conserved in many *Drosophila *species, in which a specific TRA-2 isoform represses splicing of the M1 intron in the TRA-2 pre-mRNA by binding to an intronic splicing silencer (CAAGR; TRA-2-ISS). Interestingly, the search in *Aeadsx *for putative RBP1 and TRA-2-ISS binding elements was successful, revealing their presence as a main cluster in the intronic region upstream of the female-specific exon 5a (Figure 6).

The presence of all these conserved regulatory elements suggests that TRA-like, TRA-2 and RBP1 conserved proteins could be involved in *Ae. aegypti *as upstream *dsx *splicing regulators, as in *Drosophila*. As previously reported for *Anopheles gambiae *[55], *tra-2 *and *rbp1 *putative orthologs are also present in the *Aedes *genome. We failed to find clear BLAST hits for TRA homologous proteins in *Aedes *or *Anopheles*, probably because of the very weak sequence conservation of TRA, which has been well documented in dipteran species and in Hymenopterans [9,13,14,49,56,57].

In Table S2 of additional file 4, the sequences of all identified *Aedes aegypti dsx *repeat elements and their counterparts in *Drosophila*, *Anopheles *and *Nasonia *are summarized.

### Evolution of splicing regulation in *Aeadsx*

Our analysis revealed that a bipartite distribution of putative *cis*-acting elements is present between the two female-specific *Aeadsx *exons, with TRA-2-ISS, RBP1 binding sites and *Nvdsx*RE almost exclusively present in the "strong" exon 5a and in the upstream intron, and putative *Aedes *TRA/TRA-2 binding sites and the PRE element present only in the "weak" exon 5b. The analysis of acceptor and donor splicing sites of the *Aeadsx *sex-specific region revealed that 3' and 5' splicing sites flanking exon 5a ("strong") are apparently optimal, while 3' and 5' splicing sites flanking exon 5b ("weak") strongly deviate from consensus. These findings suggest that in *Aedes aegypti*, the regulation of the sex-specific splicing of *dsx *is achieved by a different mechanism than in *Drosophila *and other dipteran species, using at least two different splicing regulators acting on the two alternatively used female-specific exons.

Our study show that splicing regulation of *dsx *is not as robustly conserved as previously described in other dipteran species, that that there is a bipartite distribution of putative cis-regulatory elements in the female-specific exons of *Aeadsx*, and that some of these putative *cis *elements are also present in the *dsx *of hymenopteran species. Based on our study we propose that the upstream splicing regulators of *Aeadsx *splicing act in females as activators of the weak female-specific exon 5b, while simultaneously repressing the utilization of the first female-specific exon 5a in a subset of *Aeadsx *pre-mRNAs. In the other *Aeadsx *mRNAs female-specific exon 5a is also included in the mature transcript, because this repression does not take place constitutively or very efficiently. We propose that in males, both exons 5a and 5b are skipped out of the mRNA by splicing factors able to inhibit the use of the corresponding strong splice site in exon 5a.

We propose a model of *Aeadsx *regulation in which, similarly to *Drosophila *and other dipteran species, the use of the conserved female-specific exon 5b is enhanced in females by a splicing activator, while a splicing repressor acts on exon 5a. A female-specific SR-F protein (a TRA-like protein) would be required in females to promote the alternative inclusion of female-specific exon 5b through the TRA/TRA-2 elements, while "the strong" exon 5a would be included by default; a male-specific SR-M would be required in males to repress the inclusion of the "strong" exon 5a possibly by TRA-2-ISS elements and *Nvdsx*RE, while exon 5b would be excluded because of the absence of the SR-F protein, leading to the *dsx *male-specific splicing. The splicing repression of exon 5a could be achieved by a direct action of the male-determining gene, which would encode a splicing factor in this case.

We suggest that the peculiar evolvability of *dsx *splicing regulation in *Aedes *could be an indication that, differently to other dipteran species, the primary signal M acts directly on *dsx *in this mosquito species, which have homomorphic sexual chromosomes and hence a "young" recently recruited Y chromosome bearing the M. The presence of multiple copies of the *Drosophila *TRA/TRA-2 binding element and the TRA-2-ISS element in exon 5a and its upstream intron sequence suggests an involvement of a putative *Aedes *TRA-2 as a cofactor of SR splicing regulators (possibly including the male-determining factor) in the regulation of *dsx *splicing. In the *Aedes aegypti *genome, there are three putative *tra-2 *orthologs that could be involved in this process, either alone or in combination.

Less clear is the reason for the presence of the *Nvdsx*RE element. These elements are completely different from the *Drosophila *consensus and hence are not expected to be targets of TRA/TRA-2 orthologous proteins but rather of other splicing factors. Perhaps these are additional splicing signals required to evolutionarily stabilize the *dsx *sex-specific splicing, which appears to be absent in *Drosophila *and *Anopheles dsx *female-specific exons.

**Figure 7:**
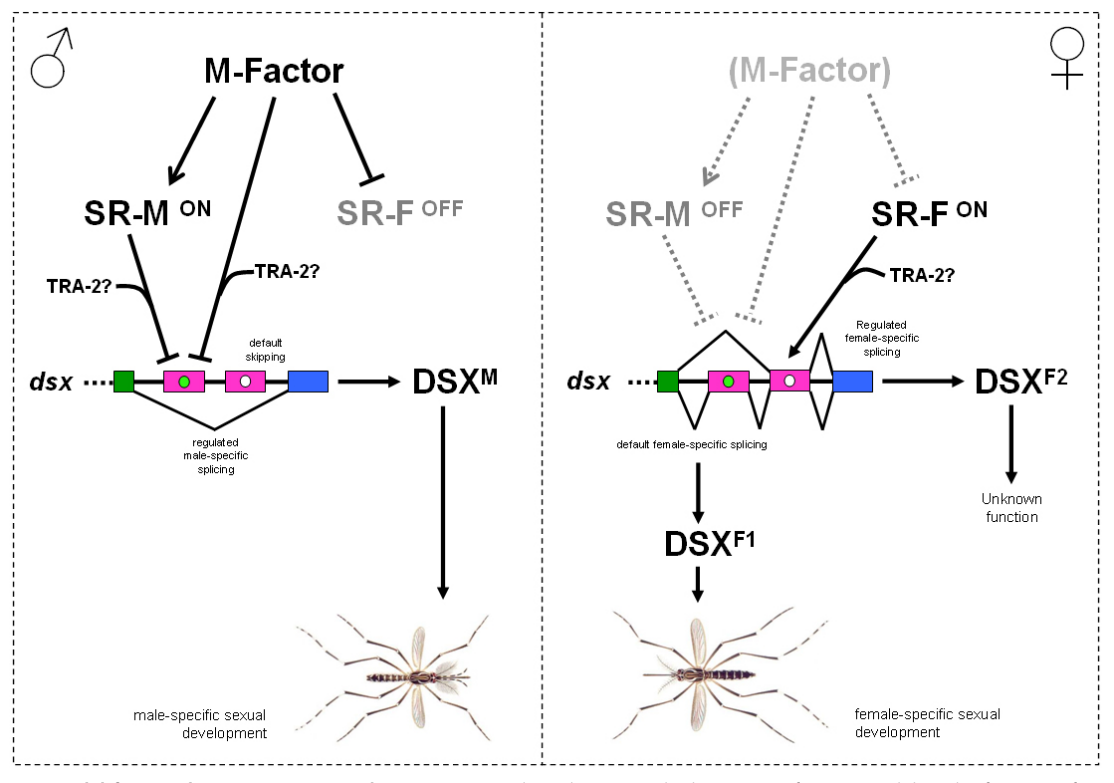
**Model for sex determination in *Aedes aegypti***. In male embryos a male-determining factor (*M*) inhibits the function of an SR-F protein required for the female-specific splicing of exon 5b and activates the function of an SR-M protein required for the male-specific repression of exon 5a. It is also conceivable that the *M *could directly control the splicing of *Aeadsx *exon 5a. In any case, the result is the skipping of both female-specific exons and only a male-specific product of *dsx *gene is produced in male embryos and this product induces the male development. In female embryos the absence of the *M *leads to the default splicing of female-specific exons 5a and 5b and to the activation of the SR-F factor which in turn regulates the female-specific splicing of exon 5b. As a result two female-specific DSX proteins are produced that induce female development. Alternative male-specific and female-specific exons are represented as blue boxes and pink boxes, respectively. Green dot represent *Nvdsx*RE and TRA-2-ISS elements. White dot represents TRA/TRA-2 binding sites.

We propose a model for sex determination in *Aedes aegypti *in which the male-determining factor M acts directly or indirectly either to activate a downstream SR-M factor or to encode the SR-M itself or finally to inhibit an SR-F factor. Two alternative parallel regulatory events in both sexes lead alternatively to the *Aeadsx *male-specific or female-specific splicing (Figure 7).

In *Apis mellifera*, Cristino *et al. *[22] reported that honeybee *dsx *is sex-specifically spliced, indicating that the use of different splicing forms of DSX in controlling sexual differentiation was present in the common ancestor of holometabolous insects. In *Apis *sex-specific splicing regulation shares similar features of both *B. mori *splicing regulation (female-specific exon skipping) and *Drosophila *splicing regulation (sex-specific 3' splice site). Based on a further *Apis dsx *study Cho *et al. *[21] proposed that the female *dsx *form is the default (as observed in the silk moth *Bombyx dsx*) in ancestral holometabolous insects and that the male form is generated by suppressing the female-specific splicing. On the basis of the parsimony principle, they inferred from the above observations the following: (1) the common ancestor of holometabolous insects had both the dipteran-type and lepidopteran-type sex-specific *dsx *splicing patterns, (2) hymenopterans retain both ancestral patterns, and (3) dipterans and lepidopterans each retain either one or the other of the ancestral patterns.

The *Aedes dsx *gene contains not only the *Drosophila *TRA/TRA-2 repeats but also hymenopteran-like *dsx*RE repeats and seems to have a female-specific default splicing. This is similar to the regulation of *Apis dsx *gene but different from other dipteran species including the other mosquito *Anopheles gambiae*. Hence, the ancestral *dsx *splicing regulation observed in *Apis *(and in lepidopteran) seems to have been stably maintained in *Aedes aegypti*, which constitutes the first dipteran species reported with this type of splicing regulation.

## Conclusion

As in other dipteran species, *dsx *seems to be involved in the control of sexual differentiation in *Aedes *mosquito based on its conservation of sex-specific expression. In contrast to other dipteran orthologs, the *Aeadsx *gene produces a novel female-specific isoform of DM-domain containing transcription factor differing in the C-terminus. The novel female-specific isoform has the long common region (248 aa) present in the other two sex-specific isoforms and a short different C-terminus (18 aa). *Aeadsx *gene exhibits more complex sex-specific splicing regulation (an exon skipping in males, exon skipping in females, intron retention in both sexes and two potential alternative polyadenylation sites) that seems to be a combination of dipteran and hymenopteran types of *dsx *regulation.

Our study show that 1) *dsx *splicing regulation is slightly less conserved then previously described in dipteran species, including the other mosquito *Anopheles gambiae*, 2) there is a bipartite distribution of putative *cis *regulatory elements in the two alternatively regulated female-specific *Aeadsx *exons, 3) the putative *cis *elements described in the *dsx *of dipteran and hymenopteran species are present also in untranslated regions of the *Aedes dsx *gene.

These findings led us to propose that the upstream splicing regulators of *dsx *splicing seem to act A) in females as activators of the weak female-specific exon 5b, which simultaneously causes the skipping of the first female-specific strong exon 5a in a subset of *Aeadsx *pre-mRNA. In the remaining *Aeadsx *pre-mRNA molecules a default splicing event include the strong female-specific exon 5a in the mature transcript; B) in males as repressors of the female-specific strong exon 5a, where the weak female-specific exon 5b is spliced out by a default mechanism.

The evolution of these differences in the splicing regulation of the *dsx *orthologs of two mosquitoes, *Anopheles *and *Aedes*, could be explained by a direct involvement of different primary signals that evolved upstream of *dsx*, namely the Y-linked *M *factor in *Anopheles *and the *M/m *male-determining locus on one (neo Y) of the *Aedes *sexual homomorphic chromosomes, overcoming the use of an intermediate "transducer" such as *transformer*, although widely conserved in insects [58].

The identification of female- and male-specific transcripts of *Aeadsx *represents an important step toward the understanding of the sex differentiation process in *Ae. aegypti*. This genomic analysis and the identification of novel putative *cis*-acting elements will facilitate the isolation of upstream regulators, which are still unidentified despite a sequenced genome, also for *Anopheles dsx *orthologue. Recently, transcription profiles of adult female and male *Aedes *mosquitoes were defined through microarray analysis, leading to the identification of 669 and 635 transcripts enriched in females and males, respectively [26]. The differential expression of hundreds of these genes, which could be involved in controlling the phenotypic and molecular sexual dimorphisms of *Aedes aegypti*, could be directly achieved by AeaDSX isoforms. In future, *in vivo *knockdown experiments by RNAi against the three sex-specific *Aedes *DSX isoforms will confirm this hypothesis and will led to the identification of new DSX targets in insects.

Furthermore, this knowledge may result in the development of novel approaches for vector-targeted control of disease, as recently shown by the isolation and use of sex-specifically expressed genes to develop transgenic sexing strains in *Aedes aegypti*, which are useful for the male sterile insect technique [59].

## Methods

### *Aeadsx *cloning strategy

Our starting point was the genome sequence of *Ae. aegypti *AaegL1 [26]. We performed a BLASTP search of the Ensembl *Ae. aegypti *genomic database (http://www.ensembl.org/) using the non-sex-specific region of *Anopheles gambiae *DSX protein (236 aa) as a virtual probe, and we identified a draft sequence located in supercontig 1.370 encoding a putative protein region 68% identical to the corresponding region encoded by *An. gambiae dsx *(*Angdsx*). In *Angdsx*, sex-specific transcripts share the same 3' UTR sequence [19]. This is in contrast to *Drosophila*, in which *dsx *sex-specific transcripts terminate with different 3' UTR sequences [5,7]. Assuming that the *Aeadsx *homologue shares the same 3' UTR regulation of *An. gambiae*, we used a male-specific nucleotide sequence derived from an *Angdsx *cDNA clone (*Angdsx*^*M*^, kindly provided by Pannuti A. and Lucchesi J., Emory University - USA) to search via the BLASTN matching function of an *Ae. aegypti *EST database (http://aaegypti.vectorbase.org/). We identified a cDNA sequence (NABNG41TR, brugia_4289 of 537 bp) showing 59% identity at the nucleotide level (166/282). Using specific primer pairs for this EST clone (dsx5 primer) and for the genomic sequence previously identified *in silico *encoding the N-terminal region of the putative *Aedes *DSX protein (dsx1 primer), we performed an RT-PCR analysis on RNA samples extracted from adult sexed *Ae. aegypti *mosquitoes and successfully amplified sex-specific products of the *Aeadsx *gene (two female-specific and one male-specific cDNAs).

### RT-PCR analyses

Total RNA was extracted using the TRIzol Reagent (Invitrogen) from the following developmental stages: E1 = 0-1.5 h embryos; E2 = 1.5-2 h embryos; E3 = 2-5 h embryos; E4 = 8-12 h embryos; E = 0-36 h embryos; O = dissected ovaries; FC = female carcasses depleted of ovaries; L^12^= early larvae; L^34^= late larvae; P = pupae; M = adult males; F = adult female. All samples except for M, F, O and FC are constituted by mixed sexes. Aliquots of 1 μg of each RNA were treated with RNase-free DNase I, Amplification Grade (Invitrogen), and first-strand cDNAs were synthesized by Superscript First-Strand Synthesis System (Invitrogen) according to the manufacturer's instruction. One twentieth of the cDNA template was used in 49-μl PCRs containing primer pairs specific for the various cDNAs, 50 mM KCl, 10 mM Tris^·^HCl (pH 8.3), 1.5 mM MgCl_2_, 1 μM each primer, 200 μM dNTPs (Roche), and 2.5 units *Taq *DNA polymerase (Roche). Appropriate annealing temperatures and cycle numbers were adjusted to individual primers. Positive controls and standardization were performed with the following primers:

Aearp49+ (5'-CCAAGATCGTCAAGAAGCGG-3')

Aearp49- (5'-GGTTGGTCACAGCGATGG-3').

These primers amplify the constitutive ribosomal protein 49 of *Aedes aegypti*. Amplifications were performed by stopping the PCR reaction after 25 cycles, 30 cycles, 35 cycles and 40 cycles. The amplification products were analyzed on 1% agarose gels. Following 30 cycles of RT-PCR amplification, we observed no saturation in the intensity of the Aearp49 amplification fragment.

Other *Aeadsx*-specific primers included:

dsx1: 5'-GAACGCCGCCGAACTGTG-3'

dsx2: 5'-ATCTTCGGTGCTGGGACAG-3'

dsx3: 5'-GATACTGAAAGGCGCCGACG-3'

dsx4: 5'-ATTGTGTGTCCAACCTCATTTC-3'

dsx5: 5'-GCAGAATATGGGACTGGTGC-3'.

The amplification products were cloned into the pGEMT-Easy Vector (Promega) according to manufacturer's instructions.

### Rapid amplification of cDNA ends (RACE) and sequence analyses

The 5'- and 3 '-ends of the *Aeadsx *cDNAs were determined with the Smart Race Amplification kit (Clontech Laboratories). Reverse transcription was performed as recommended by the supplier. cDNAs containing open reading frames (ORFs) were cloned into the pGEMT-Easy Vector (Promega) and sequenced with the Applied Biosystem BigDye 1.1 sequencing kit. Sequence alignments were performed with the Clustal-W software. The following *Ae. aegypti *cDNA sequence files were deposited in GenBank: Aeadsx^M1 ^[GenBank: DQ440534], Aeadsx^F1 ^[GenBank: DQ440532] and Aeadsx^F2 ^[GenBank: DQ440533].

### Northern blot analysis

Twenty micrograms of total RNA from adult sexed mosquitoes was isolated as described above and blotted onto nylon membranes (Hybond NX - Amersham Pharmacia). Hybridizations were performed at 42°C overnight in a buffer of 50% formamide, 6× SSC, 5× Denhardt, 1% SDS and 100 μg/ml salmon sperm DNA. Following hybridizations, the filters were washed for 10 minutes at room temperature in 2× SSC and 0.1% SDS, followed by two washes for 20 minutes each at 42°C in 1× SSC and 0.1% SDS, and finally one wash for 15 minutes at room temperature in 1× SSC and 0.1% SDS. The female-specific probe (400 bp) was prepared by nick-translation (Invitrogen) labeling of *Aeadsx *cDNA sequences in the presence of [α-^32^P]dCTP (3,000 Ci/mmol Amersham). Filters were exposed on Kodak X-OMAT Scientific imaging film.

### Phylogenetic and Molecular Evolutionary Analyses

The nucleotide sequences encoding the OD1 domain and the OD2 domain of the following dipteran *doublesex *homologues (*Drosophila melanogaster *[GenBank: ABK30895], *Ceratitis capitata *[GenBank: AAN63598], *Bactrocera tryoni *[GenBank: AAB99947], *B. correcta *[GenBank: ACN73403], *B. dorsalis *[GenBank: AAV85891], *B. oleae *[GenBank: CAD67986], *Anastrepha bistrigata *[GenBank: ABF50960], *A. serpentina *[GenBank: ABF50957], *A. amita *[GenBank: ABF50961], *A. fraterculus *[GenBank: ABF50955], *Anopheles gambiae *[GenBank: AAZ78363], *Aedes aegypti *[GenBank: ABD96571], *Musca domestica *[GenBank: AAR23812], *Lucilia cuprina *[GenBank: ADG37649] and *Megaselia scalaris *[GenBank: AAK38831]) were aligned using Clustal-W, and the resulting alignment [60] was manually edited using BioEdit v7.0.5.3. The maximum parsimony and neighbor-joining trees were obtained using the MEGA 5 package [61]. The reliability of the resulting topologies was tested by the bootstrap method [62]. Bootstrap proportions were calculated by analysis of 1500 replicates. Phylogenies were rooted using *dsx *OD1 and OD2 domains of Lepidoptera *Bombyx mori *[GenBank: NM_001043406] and *Danaus plexippus *[GenBank: EY269445]. Pairwise synonymous (dS) and non-synonymous (dN) nucleotide substitution rates were estimated using the Jukes-Cantor distance model with the Nei-Gojobori method [63] implemented in MEGA 5. The mean pairwise ratio of dN/dS was calculated and used to examine whether the sequences evolve under purifying constraint (dN/dS < 1) or positive selection for amino acid changes (dN/dS > 1).

## Authors' contributions

GS designed the general research project. GS, BA, CP, FL, MS and UM designed the experimental plans. UM performed the molecular cloning experiments. UM, MS and FL performed the *dsx *developmental expression analyses, and MS performed the *Aearp49 *developmental expression analysis. UM and MS performed *in sili*co analyses to define the *dsx *genomic organization. AM and VZ helped with the molecular biology experiments. MS and GS planned comparative analyses to identify *cis*-regulatory elements potentially involved in the *dsx *splicing regulation. MS performed bioinformatic, phylogenetic and molecular evolutionary analyses. UM, MS, AM, VZ, FL, BA, CP and GS analyzed and discussed data. MS and GS wrote the paper. All authors read and approved the final manuscript.

## Supplementary Material

Additional file 1 **Table S1 Modified Censor output table of repetitive elements identified in *Aeadsx *intronic regions**. Click here for file

Additional file 2 **Figure S1 Censor analysis on *Aeadsx *introns**. Censor software graphically maps detected repeats with color-coding of different types of repeats. Legend of Censor output is available at: http://www.girinst.org/censor/help.html#MAP. Click here for file

Additional file 3 **Figure S3 Microsynteny analysis**. Syntenic *dsx*-containing regions of *Aedes aegypti *and *Anopheles gambiae *are reported. In red the position of putative genes. Click here for file

Additional file 4 **Tables S2 Putative *cis*-acting elements identified in *Aeadsx *and homologous elements in *D. melanogaster*, *An. gambiae *and *N. vitripennis***. Bold letters indicate identical positions respect to the sequences of *Drosophila *or *Nasonia *elements. Click here for file
