# Supplementary material for: Genomic organization and splicing evolution of the doublesex gene, a Drosophila regulator of sexual differentiation, in the dengue and yellow fever mosquito Aedes aegypti
Source: BMC Evol Biol. 2011 Feb 10;11:41. doi: 10.1186/1471-2148-11-41 (PMC3045327; doi:10.1186/1471-2148-11-41)
Supplement: Additional file 1 — Table S1 Modified Censor output table of repetitive elements identified in Aeadsx intronic regions. [file 1471-2148-11-41-S1.PDF]

Table S1 - Modified Censor output of repetitive elements identified in *Aeadsx* intronic regions

Intron	Name	Class	From	To	Score
2	Academ-1_HM	DNA/Academ	166943	167029	238
2	ACROBAT1	DNA	199310	199381	229
2	AMPLICON_AA	NonLTR/RTE	127744	127966	873
3	AMPLICON_AA	NonLTR/RTE	30608	31008	1825
3	AMPLICON_AA	NonLTR/RTE	31342	31447	536
4	AMPLICON_AA	NonLTR/RTE	24674	24861	782
8	AMPLICON_AA	NonLTR/RTE	142	396	982
2	ARNOLD1	DNA	161461	161539	254
3	ATCOPIA8BI	LTR/Copia	37444	37483	242
2	ATHILA5_LTR	LTR/Gypsy	253795	253867	226
2	ATREP13	DNA/Helitron	91947	91994	225
2	ATREP13	DNA/Helitron	93562	93609	225
2	ATREP13	DNA/Helitron	94602	94649	225
8	ATREP9	DNA/Helitron	14534	14576	226
8	Baggins1_Cis	Interspersed_Repeat	6003	6029	220
4	BEL10-I_AG	LTR/BEL	80606	80640	227
4	BEL1-I_DV	LTR/BEL	81832	82013	289
7	BEL-2-I_XT	LTR/BEL	5558	5610	289
4	BEL4-I_AG	LTR/BEL	80758	80823	249
7	BEL5-I_AG	LTR/BEL	5952	6354	499
2	BGLII_B2	ERV/ERV2	111961	112128	343
2	BHIKHARI-4-LTR_DR	LTR	123476	123566	296
8	CACTA-N	DNA/EnSpm	5635	5702	226
2	CASINA	DNA	242606	242649	238
2	CER15-1-I_CE	LTR	152256	152466	239
2	CER15-1-I_CE	LTR	153088	153686	563
2	CER8-I_CE	LTR	68542	68793	288
2	CER8-I_CE	LTR	172349	172529	345
2	CER8-I_CE	LTR	172558	172680	210
2	CER8-I_CE	LTR	177704	177954	438
2	CER8-I_CE	LTR	195554	195627	227
2	CER8-I_CE	LTR	196048	196113	219
8	CER8-I_CE	LTR	13181	13454	335
2	Chapaev-3_HM	DNA/Chapaev	83454	83494	202
2	Chapaev3-1_AA	DNA/Chapaev	6417	6632	1395
2	Chapaev3-1_AA	DNA/Chapaev	41302	41331	219
2	Chapaev3-1_AA	DNA/Chapaev	41332	41684	1866
2	Chapaev3-1_AA	DNA/Chapaev	171205	171544	1313
2	Chapaev3-1_AA	DNA/Chapaev	171685	172032	1220
2	Chapaev3-1_AA	DNA/Chapaev	232910	233032	406
2	Chapaev3-1_AA	DNA/Chapaev	237701	237847	592
2	Chapaev3-1_AA	DNA/Chapaev	272329	272538	1236
2	Chapaev3-1_AA	DNA/Chapaev	272539	272693	1144
4	Chapaev3-1_AA	DNA/Chapaev	52773	52887	228
4	Chapaev3-1_AA	DNA/Chapaev	67248	67398	607
4	Chapaev3-1_AA	DNA/Chapaev	67446	67515	330
5	Chapaev3-1_AA	DNA/Chapaev	3876	4050	815
5	Chapaev3-1_AA	DNA/Chapaev	4058	4113	286
2	Chapaev3-3_AA	DNA/Chapaev	44430	44598	462
2	Chapaev3-3_AA	DNA/Chapaev	247011	247131	363
3	Chapaev3-3_AA	DNA/Chapaev	23320	23459	372
8	Chapaev3-6_HM	DNA/Chapaev	9109	9160	237
4	Chapaev3-N1_HR	DNA/Chapaev	46368	46428	234
2	CHARLIE1	DNA/hAT	265040	265103	352
2	Copia1-VV_LTR	LTR/Copia	103682	103726	216
2	Copia23-VV_I	LTR/Copia	229292	229339	203
2	Copia-26_DPu-I	LTR	122470	122606	214
2	Copia-26_DPu-I	LTR	122636	122787	226
2	Copia-26_DPu-I	LTR	123259	123387	274
2	Copia-26_DPu-I	LTR	123876	123983	223
2	Copia-26_DPu-I	LTR	124071	124201	241
2	Copia-26_DPu-I	LTR	180604	180726	234
2	Copia-26_DPu-I	LTR	194816	194929	227

2	Copia-26_DPu-I	LTR	194936	195060	239
2	Copia-26_DPu-I	LTR	195203	195356	276
2	Copia-26_DPu-I	LTR	196378	196481	212
2	Copia-26_DPu-I	LTR	200322	200477	226
2	Copia-26_DPu-I	LTR	200528	200671	343
2	Copia-26_DPu-I	LTR	200909	201030	223
3	COPIA4-I_AG	LTR/Copia	17615	17794	305
3	Copia53-PTR_I	LTR/Copia	17427	17586	350
2	Copia-85_SB-I	LTR/Copia	127580	127634	242
3	Copia9-NVi_I	LTR/Copia	18180	18266	314
2	CR1-13_HM	NonLTR/CR1	144600	144651	202
2	CR1-15_HM	NonLTR/CR1	202545	202629	206
2	CR1-20_HM	NonLTR/CR1	258485	258636	209
2	CR1-24_HM	NonLTR/CR1	167298	167387	256
2	CR1-25_BF	NonLTR/CR1	63113	63156	221
2	CR1-3_HM	NonLTR/CR1	4389	4482	226
2	CR1-4_AG	NonLTR/CR1	84917	84984	202
2	CR1-4_HM	NonLTR/CR1	193778	193817	200
2	CR1-45_HM	NonLTR/CR1	1443	1527	220
2	CR1-67_HM	NonLTR/CR1	175872	175936	217
4	CR1-67_HM	NonLTR/CR1	68106	68162	221
2	Crack-29_BF	NonLTR/Crack	79502	79548	235
2	Crack-3_HM	NonLTR/Crack	142020	142101	213
3	CRE2	NonLTR/CRE	8483	8590	232
3	Crypton-2_TCa	DNA/Crypton	23674	23817	260
3	DIRS-8_DR	LTR/DIRS	25911	26293	690
4	DIVER_I	LTR	84749	84796	233
4	DNA-1_Bf	DNA	4223	4337	275
2	DNA2-1_CB	DNA	42504	42629	233
2	DNA-2-1_NV	DNA	150142	150209	248
4	DNA-2-1_NV	DNA	27313	27376	228
4	DNA-2-1_NV	DNA	28084	28209	258
4	DNA-2-2_HM	DNA	15113	15159	217
4	DNA-6-N8_DR	DNA	52920	54658	680
4	DNA-8-1_SP	DNA	22234	22279	207
2	DNA-8-15_DR	DNA	270957	271006	215
2	DNA8-61_AP	DNA	120710	120792	204
2	EnSpm-1_AA	DNA	104508	104552	240
2	EnSpm-1_AA	DNA	168443	168558	345
2	EnSpm-1_AA	DNA	184568	184706	412
2	EnSpm-1_AA	DNA	232209	232251	272
4	EnSpm-1_AA	DNA	51896	51945	241
4	EnSpm-1_AA	DNA	55436	55503	261
4	EnSpm-2_BF	DNA/EnSpm	85155	85205	242
4	EnSpm2_SB	DNA/EnSpm	55779	55885	228
2	ENSPM2_VV	DNA/EnSpm	4820	4910	205
2	ENSPM2_VV	DNA/EnSpm	58395	58454	210
5	EnSpm-3_HM	DNA/EnSpm	4460	4537	208
2	EnSpm-3_VV	DNA/EnSpm	256245	256323	225
4	EnSpm-5_HV	DNA/EnSpm	43028	43087	219
2	EnSpm-N14_SBi	DNA/EnSpm	189631	189672	203
2	EnSpm-N2_SBi	DNA/EnSpm	115372	115412	254
2	ERE1_EH	Interspersed_Repeat	15324	15520	245
3	ERE2_EH	Interspersed_Repeat	26691	26796	210
2	ERV2-2_Opr-I	ERV/ERV2	250882	250935	245
2	ERV2X1A-LTR_ML	ERV/ERV2	68881	68997	274
2	ERV2X1A-LTR_ML	ERV/ERV2	69780	69885	265
2	ERV2X1A-LTR_ML	ERV/ERV2	69924	70031	243
2	ERV2X1A-LTR_ML	ERV/ERV2	70082	70129	206
2	ERV2X1A-LTR_ML	ERV/ERV2	70154	70242	226
2	ERV2X1A-LTR_ML	ERV/ERV2	70691	70796	270
2	ERV2X1A-LTR_ML	ERV/ERV2	71005	71085	247
2	FB4_DM	DNA/Mariner	7533	7571	234
2	FEILAI_AA	NonLTR/SINE	20324	20357	249
2	FEILAI_AA	NonLTR/SINE	20358	20391	240
2	FEILAI_AA	NonLTR/SINE	20392	20506	858
2	FEILAI_AA	NonLTR/SINE	42955	43175	1416
2	FEILAI_AA	NonLTR/SINE	49995	50161	1261
2	FEILAI_AA	NonLTR/SINE	50163	50218	481
2	FEILAI_AA	NonLTR/SINE	50891	51020	289

2	FEILAI_AA	NonLTR/SINE	64781	64827	312
2	FEILAI_AA	NonLTR/SINE	73940	74062	919
2	FEILAI_AA	NonLTR/SINE	77372	77456	597
2	FEILAI_AA	NonLTR/SINE	143656	143921	1467
2	FEILAI_AA	NonLTR/SINE	169851	170064	1588
2	FEILAI_AA	NonLTR/SINE	170571	170616	331
2	FEILAI_AA	NonLTR/SINE	198470	198630	1255
2	FEILAI_AA	NonLTR/SINE	202201	202486	2289
2	FEILAI_AA	NonLTR/SINE	217436	217667	1227
2	FEILAI_AA	NonLTR/SINE	241767	242050	2345
2	FEILAI_AA	NonLTR/SINE	250172	250287	614
3	FEILAI_AA	NonLTR/SINE	27554	27838	2202
4	FEILAI_AA	NonLTR/SINE	29240	29518	2039
4	FEILAI_AA	NonLTR/SINE	41750	42037	2417
4	FEILAI_AA	NonLTR/SINE	61040	61326	2360
4	FEILAI_AA	NonLTR/SINE	62690	62759	327
4	FEILAI_AA	NonLTR/SINE	64116	64385	1927
4	FEILAI_AA	NonLTR/SINE	65973	66059	267
4	FEILAI_AA	NonLTR/SINE	72334	72386	251
4	FEILAI_AA	NonLTR/SINE	72387	72573	1159
5	FEILAI_AA	NonLTR/SINE	1760	2044	2038
8	FEILAI_AA	NonLTR/SINE	10830	11116	2397
8	FEILAI_AA	NonLTR/SINE	17444	17584	1003
3	Gecko	NonLTR/SINE/SINE2	13511	13553	247
4	Gecko	NonLTR/SINE/SINE2	55293	55434	849
4	Ginger1-1_AP	DNA/Ginger1	29839	29931	202
5	Ginger1-6_HM	DNA/Ginger1	12769	12848	223
3	Ginger1-9_HM	DNA/Ginger1	5182	5342	240
2	GLT2_SM	LTR	111598	111648	203
2	GmCOPIA10_I	LTR/Copia	210516	210549	220
2	Gypsy10-LTR_SP	LTR/Gypsy	79654	79704	234
2	Gypsy14-NVi_I	LTR/Gypsy	99494	99537	223
4	Gypsy15-VV_I	LTR/Gypsy	68497	68550	246
2	Gypsy18-VV_LTR	LTR/Gypsy	218835	218965	200
2	Gypsy1-PP_LTR	LTR/Gypsy	90703	90801	231
2	Gypsy1-PP_LTR	LTR/Gypsy	96842	96956	211
2	Gypsy1-PP_LTR	LTR/Gypsy	148044	148178	203
2	Gypsy1-PP_LTR	LTR/Gypsy	149546	149656	200
2	Gypsy1-PP_LTR	LTR/Gypsy	151117	151250	226
2	Gypsy1-PP_LTR	LTR/Gypsy	151618	151710	279
2	Gypsy1-PP_LTR	LTR/Gypsy	195646	195828	333
2	Gypsy1-PP_LTR	LTR/Gypsy	195853	196035	307
2	Gypsy1-PP_LTR	LTR/Gypsy	197140	197224	208
2	Gypsy1-PP_LTR	LTR/Gypsy	256335	256464	231
2	Gypsy1-PP_LTR	LTR/Gypsy	261081	261250	231
2	Gypsy1-PP_LTR	LTR/Gypsy	261649	261761	237
2	Gypsy-22_BD-I	LTR/Gypsy	223140	223205	225
2	Gypsy22-VV_I	LTR/Gypsy	56730	56812	209
2	Gypsy22-VV_LTR	LTR/Gypsy	214575	214646	209
2	Gypsy-37-I_NV	LTR/Gypsy	260724	260777	248
4	GYPSY48-I_AG	LTR/Gypsy	19907	19964	239
2	Gypsy4-I_Dmoj	LTR/Gypsy	186797	186871	233
2	Gypsy4-SM_I	LTR/Gypsy	223790	223847	213
2	Gypsy4-VV_I	LTR/Gypsy	73670	73725	225
2	Gypsy63-I_DR	LTR/Gypsy	262491	262547	244
2	Gypsy-72-LTR_ZM	LTR/Gypsy	196531	196713	428
2	Gypsy7-I_DR	LTR/Gypsy	140660	140720	237
2	Gypsy-80-I_ZM	LTR/Gypsy	121031	121091	239
4	Gypsy86-I_DR	LTR/Gypsy	29727	29778	215
2	Gypsy9-SM_LTR	LTR/Gypsy	259265	259475	270
2	GYVIT1_I	LTR/Gypsy	198407	198439	218
4	HARB-8_SBi	DNA/EnSpm	477	652	243
4	Harbinger-3_XT	DNA/Harbinger	37259	37301	230
2	hAT-1_PTr	DNA/hAT	31044	31078	216
3	hAT1-2_NV	DNA/hAT	3222	3302	252
2	hAT-12_XT	DNA/hAT	205190	205286	252
2	hAT-13_HM	DNA/hAT	240915	240987	233
2	hAT-13_HM	DNA/hAT	257999	258077	227
2	HAT15_CB	DNA/hAT	225595	225724	217
3	HAT15_CB	DNA/hAT	7449	7538	250

8	HAT15_CB	DNA/hAT	10568	10710	361
2	hAT-2_AG	DNA/hAT	57447	57513	205
2	hAT-2_HM	DNA/hAT	208745	208846	240
4	hAT-27_SM	DNA/hAT	85653	85736	216
2	hAT-3_PTr	DNA/hAT	119957	120036	236
5	hAT-3_PTr	DNA/hAT	7716	7783	231
2	hAT-42_SM	DNA/hAT	132300	132367	209
2	hAT-46_HM	DNA/hAT	46234	46274	230
2	hAT-70_HM	DNA/hAT	5037	5121	275
4	hAT-70_HM	DNA/hAT	65451	65485	224
2	hAT-75_HM	DNA/hAT	78896	79031	217
2	hATm-1_AA	DNA/hAT	215164	215214	257
2	hATm-2_AA	DNA/hAT	10038	10100	340
2	hATm-2_AA	DNA/hAT	59704	59742	222
2	hATm-2_AA	DNA/hAT	62814	62881	225
2	hATm-2_AA	DNA/hAT	164639	164691	233
2	hATm-2_AA	DNA/hAT	224042	224150	208
2	hATm-2_AA	DNA/hAT	248298	248649	584
4	hATm-2_AA	DNA/hAT	77654	77729	236
7	hATm-3_AA	DNA/hAT	536	719	940
2	hATm-49_HM	DNA/hAT	104944	104995	217
2	hAT-N22_DR	DNA/hAT	112803	113641	680
2	HATSOD1	DNA/hAT	60725	60762	229
2	hATx-20_SM	DNA/hAT	51702	51775	327
2	hATx-20_SM	DNA/hAT	53058	53167	398
2	hATx-4_HM	DNA/hAT	52542	52606	253
2	hATx-4_SM	DNA/hAT	111811	111857	236
5	HELITRON1_CE	DNA/Helitron	5814	5988	268
8	HELITRON1_CE	DNA/Helitron	10760	10800	206
2	Helitron-1_DR	DNA/Helitron	239716	239802	212
4	Helitron-1_HM	DNA/Helitron	83502	83554	217
2	Helitron-1_NVi	DNA/Helitron	144539	144580	212
4	Helitron-2_HM	DNA/Helitron	75517	75615	251
2	HELITRON3_CB	DNA/Helitron	181085	181177	274
4	Helitron-6_NVi	DNA/Helitron	59078	59125	209
2	HELITRON7_CB	DNA/Helitron	163788	163987	211
2	HELITRON7_CB	DNA/Helitron	172956	173540	568
2	HELITRON7_CB	DNA/Helitron	173557	174110	837
2	HELITRON7_CB	DNA/Helitron	174491	175032	539
4	HELITRON7_CB	DNA/Helitron	2547	3016	399
4	HELITRON7_CB	DNA/Helitron	3187	3660	508
4	HELITRON7_CB	DNA/Helitron	26099	26198	229
4	HELITRON7_CB	DNA/Helitron	27636	28060	422
4	HELITRON7_CB	DNA/Helitron	28395	28702	292
4	HELITRON7_CB	DNA/Helitron	28744	29144	374
8	HELITRON7_CB	DNA/Helitron	9891	10266	425
4	Helitron-7_NVi	DNA/Helitron	65839	65887	237
4	Helitron-N2_DR	DNA/Helitron	49929	50017	238
2	HELITRONY1_CE	DNA/Helitron	82572	82824	292
2	HELITRONY1_CE	DNA/Helitron	122124	122448	246
4	HELITRONY1_CE	DNA/Helitron	25417	25533	248
5	HELITRONY1_CE	DNA/Helitron	6730	6981	290
2	HELITRONY1A_CE	DNA/Helitron	47670	47789	293
2	HELITRONY1A_CE	DNA/Helitron	123583	123823	427
2	HELITRONY1A_CE	DNA/Helitron	179574	179655	252
2	HELITRONY1A_CE	DNA/Helitron	196937	197066	332
4	HELITRONY1A_CE	DNA/Helitron	26235	26518	270
8	HELITRONY1A_CE	DNA/Helitron	11258	11417	269
2	HELITRONY4_CE	DNA/Helitron	202735	202940	218
2	HELITRONY4_CE	DNA/Helitron	203464	203568	237
2	HELITRONY4_CE	DNA/Helitron	256559	256751	381
2	HELITRONY4_CE	DNA/Helitron	256945	257496	308
3	HELITRONY4_CE	DNA/Helitron	3792	4057	220
3	HELITRONY4_CE	DNA/Helitron	4126	4629	405
4	HELITRONY4_CE	DNA/Helitron	85504	85599	251
3	HELMET2	DNA/Helitron	25233	25324	224
3	HERVK22I	ERV/ERV2	2155	2202	237
3	HERVK22I	ERV/ERV2	2870	2926	245
2	HERVP71A_I	ERV	160156	160277	262
2	IS4EU-1_NV	DNA/ISL2EU	70303	70453	260

2	IS4EU-1_NV	DNA/ISL2EU	82498	82553	221
2	IS4EU-1_NV	DNA/ISL2EU	85692	85748	240
2	IS4EU-1_NV	DNA/ISL2EU	86191	86340	254
2	IS4EU-1_NV	DNA/ISL2EU	93039	93162	230
2	IS4EU-1_NV	DNA/ISL2EU	95140	95265	257
2	IS4EU-1_NV	DNA/ISL2EU	95349	95472	214
2	Jockey-1_TCa	NonLTR/Jockey	184157	184196	223
5	KenoDr1	NonLTR/Tx1	4882	4916	224
4	Kolobok-1_CB	DNA/Kolobok	11073	11109	222
2	Kolobok-1_DR	DNA/Kolobok	240233	240296	216
5	Kolobok-8_HM	DNA/Kolobok	8923	9029	220
8	L1-1_EC	NonLTR/L1	13050	13080	230
2	L1-1_SP	NonLTR/L1	40669	40723	226
2	L1-2_ME	NonLTR/L1	248145	248230	287
4	L1-2_Ttr	NonLTR/L1	68297	68433	309
2	LINE-1_AA	NonLTR/Jockey	2393	2431	206
2	LINE-1_AA	NonLTR/Jockey	2432	2580	529
2	LINE-1_AA	NonLTR/Jockey	24152	24214	542
2	LINE-1_AA	NonLTR/Jockey	24217	25045	5939
2	LINE-1_AA	NonLTR/Jockey	25046	25357	2279
2	LINE-1_AA	NonLTR/Jockey	25464	25862	3189
2	LINE-1_AA	NonLTR/Jockey	72325	72662	1481
2	LINE-1_AA	NonLTR/Jockey	72872	72932	340
2	LINE-1_AA	NonLTR/Jockey	108016	108178	772
2	LINE-1_AA	NonLTR/Jockey	138820	139294	1693
2	LINE-1_AA	NonLTR/Jockey	192146	192181	285
2	LINE-1_AA	NonLTR/Jockey	192182	192301	673
2	LINE-1_AA	NonLTR/Jockey	192306	192804	2630
2	LINE-1_AA	NonLTR/Jockey	230785	230932	666
2	LINE-1_AA	NonLTR/Jockey	233066	236901	31446
2	LINE-1_AA	NonLTR/Jockey	236902	237700	6832
2	LINE-1_AA	NonLTR/Jockey	255136	255295	662
2	LINE-1_AA	NonLTR/Jockey	264484	264770	1746
2	LINE-1_AA	NonLTR/Jockey	264784	265039	1708
2	LINE-1_AA	NonLTR/Jockey	266680	266757	431
3	LINE-1_AA	NonLTR/Jockey	28787	28861	240
3	LINE-1_AA	NonLTR/Jockey	28862	29123	1358
3	LINE-1_AA	NonLTR/Jockey	29127	29317	1112
3	LINE-1_AA	NonLTR/Jockey	38048	39388	11482
3	LINE-1_AA	NonLTR/Jockey	39722	39888	1475
4	LINE-1_AA	NonLTR/Jockey	9921	10246	858
4	LINE-1_AA	NonLTR/Jockey	20232	20261	205
4	LINE-1_AA	NonLTR/Jockey	20262	20564	1690
4	LINE-1_AA	NonLTR/Jockey	22365	22492	839
4	LINE-1_AA	NonLTR/Jockey	30935	31521	4310
4	LINE-1_AA	NonLTR/Jockey	32607	33873	5953
4	LINE-1_AA	NonLTR/Jockey	73135	73189	375
5	LINE-1_AA	NonLTR/Jockey	483	1202	3671
8	LINE-1_AA	NonLTR/Jockey	15691	15768	316
4	LINE1-13_SBi	NonLTR/L1	1909	1966	249
2	LINE1-16_ZM	NonLTR/L1	47187	47330	284
3	LINE1-27_SBi	NonLTR/L1	20437	20524	205
4	LINE1-32_ZM	NonLTR/L1	21136	21226	204
3	LINE1-34_SBi	NonLTR/L1	43048	43099	246
8	LINER1	NonLTR/I	2536	2587	295
2	LTR12B_OC	ERV/ERV2	169490	169532	235
7	LTR26_OC	ERV/ERV2	4384	4412	201
3	LTR34B_Str	ERV/ERV2	1950	2076	321
3	LTR34B_Str	ERV/ERV2	2352	2533	501
3	LTR34B_Str	ERV/ERV2	2536	2609	212
3	LTR34B_Str	ERV/ERV2	2664	2829	363
3	LTR34B_Str	ERV/ERV2	2942	3013	228
3	LTR34B_Str	ERV/ERV2	3375	3487	269
3	LTR34B_Str	ERV/ERV2	3490	3684	402
2	Mariner-20_HM	DNA/Mariner	124533	124576	222
3	Mariner-30_HM	DNA/Mariner	25506	25548	213
2	MARINER36_CB	DNA/Mariner	56524	56562	236
4	MARINER65_CB	DNA/Mariner	25222	25315	231
2	MER65I	ERV	81059	81102	210
2	MERLIN3_CB	DNA/Merlin	82251	82411	282

2	MERLIN3_CB	DNA/Merlin	85965	86072	215
2	MERLIN3_CB	DNA/Merlin	95800	95946	239
2	MERLIN3_CB	DNA/Merlin	242949	243049	231
2	MERVL_2A	ERV/ERV3	149073	149112	225
4	MINISAT2_CB	Simple/Sat/MSAT	3829	4212	362
3	MINISAT3_CB	Simple/Sat/MSAT	24696	24802	350
2	MINISAT4_CB	Simple/Sat/MSAT	177611	177689	200
2	MINISAT4_CB	Simple/Sat/MSAT	178057	178483	488
2	MINISAT4_CB	Simple/Sat/MSAT	178653	179095	406
2	MINISAT4_CB	Simple/Sat/MSAT	179896	180360	359
2	MINISAT4_CB	Simple/Sat/MSAT	180878	180982	260
2	MITE_AA	Interspersed_Repeat	37939	38243	1649
2	MITE_AA	Interspersed_Repeat	38244	38315	423
2	MITE_AA	Interspersed_Repeat	38612	38661	391
4	MITE_AA	Interspersed_Repeat	31736	32243	3921
2	MMERVK10C	ERV/ERV2	110140	110173	230
2	MOSAT-2_DR	Simple/Sat/SAT	206888	206959	407
2	Mosqul_Aa2	NonLTR	223510	223559	224
8	Mosqul_Aa2	NonLTR	5582	5631	211
3	MtPH-A6-1-Ia	DNA/Harbinger	33808	33906	224
2	MtPH-A6-2-Ia	DNA/Harbinger	46968	47008	224
2	MuDr-1_HM	DNA/MuDR	167145	167180	227
4	MuDr-1_HV	DNA/MuDR	6989	7039	269
2	MuDR1_ZM	DNA/MuDR	254685	254725	227
3	MuDR-4_VV	DNA/MuDR	527	600	211
4	MuTRI_MT	DNA/MuDR	66891	66930	220
2	NOMAD_I	LTR/Gypsy	270293	270333	224
4	Ogre-PT1_I	LTR/Gypsy	47230	47287	235
2	P-2_AP	DNA/P	118795	118841	215
2	P3_AG	DNA/P	166716	166747	219
2	P-30_HM	DNA/P	238456	238504	250
2	P-32_HM	DNA/P	4503	4539	210
3	P-8_HM	DNA/P	21907	21999	215
7	PCretro3_I	LTR/Copia	972	1035	222
4	Penelope-13_HM	NonLTR/Penelope	45243	45294	236
3	piggyBac-1N1_XT	DNA/piggyBac	19863	19897	242
7	Polinton-1_EI	DNA/Polinton	4156	4205	218
2	Polinton-2_CB	DNA/Polinton	197658	197693	222
2	Polinton-2_HM	DNA/Polinton	119259	119327	203
2	Polinton-3_HM	DNA/Polinton	247784	247837	202
4	Polinton-3_HM	DNA/Polinton	1300	1371	210
2	PONY_AA	DNA	125784	126293	3277
2	PONY_AA	DNA	140169	140659	3762
2	PONY_AA	DNA	145404	145914	3794
4	R4-1_ED	NonLTR/R4	40683	40732	206
2	R6Ag3	NonLTR	116562	116910	550
4	R7Ag2	NonLTR	43945	44150	480
8	RC123	Simple/Sat/SAT	2068	2128	219
2	RCC9	Simple/Sat/SAT	96496	96595	211
2	Rehavkus-1_DA	DNA/Rehavkus	176818	176869	202
5	Rehavkus-1_DA	DNA/Rehavkus	8775	8857	200
8	Rehavkus-1_DY	DNA/Rehavkus	10482	10566	240
2	Ricksha_a	DNA/MuDR	90111	90158	262
2	RNLTR15A2_LTR	ERV/ERV2	112611	112752	307
2	RTAg4	NonLTR	109090	109141	228
2	RTE-1_AG	NonLTR/RTE	33837	34091	504
2	RTE-2_AG	NonLTR/RTE	32131	32991	1359
2	RTE-2_AG	NonLTR/RTE	34718	35284	792
2	RTE-2_AG	NonLTR/RTE	127968	128144	707
2	RTE-2_AG	NonLTR/RTE	187695	188343	2120
2	RTE-2_AG	NonLTR/RTE	188374	189347	3036
2	RTE-2_AG	NonLTR/RTE	189791	190046	533
2	RTE-2_AG	NonLTR/RTE	190112	190609	1786
3	RTE-2_AG	NonLTR/RTE	31009	31321	715
3	RTE-2_AG	NonLTR/RTE	31462	31603	323
4	RTE-2_AG	NonLTR/RTE	24865	24996	291
4	RTE-2_AG	NonLTR/RTE	30856	30929	330
8	RTE-2_AG	NonLTR/RTE	581	718	410
8	RTE-2_AG	NonLTR/RTE	950	1022	308
4	RTE-3_AG	NonLTR/RTE	76069	76255	360

2	RTE-5_SP	NonLTR/RTE	149272	149345	266
2	Sake_BM	NonLTR/Daphne	261483	261518	204
2	SINE_TE	NonLTR/SINE	101058	101115	259
4	SMAR5	DNA/Mariner	58232	58299	202
4	Sola1-3_AA	DNA/Sola	59896	59922	257
4	Sola1-3_AA	DNA/Sola	60461	60487	257
2	Sola1-4_AA	DNA/Sola	265758	265998	598
8	Sola1-4_AA	DNA/Sola	1480	1528	294
5	Sola2-1_HM	DNA/Sola	9871	10258	559
2	Sola2-4_NVi	DNA/Sola	10425	10461	225
2	Sola3-1N1_AA	DNA/Sola	38316	38607	2323
2	Sola3-1N1_AA	DNA/Sola	58455	58709	1128
2	Sola3-1N1_AA	DNA/Sola	66254	66334	565
2	Sola3-1N1_AA	DNA/Sola	80600	80675	480
2	Sola3-1N1_AA	DNA/Sola	80686	80816	771
2	Sola3-1N1_AA	DNA/Sola	81192	81480	2453
2	Sola3-1N1_AA	DNA/Sola	89187	89233	359
2	Sola3-1N1_AA	DNA/Sola	89251	89328	502
2	Sola3-1N1_AA	DNA/Sola	162834	163067	1073
2	Sola3-1N1_AA	DNA/Sola	263184	263231	276
3	Sola3-1N1_AA	DNA/Sola	29963	30042	402
4	Sola3-1N1_AA	DNA/Sola	8816	9099	2459
4	Sola3-1N1_AA	DNA/Sola	17353	17450	297
4	Sola3-1N1_AA	DNA/Sola	21890	22046	514
4	Sola3-1N1_AA	DNA/Sola	37574	37849	2256
4	Sola3-1N1_AA	DNA/Sola	40116	40405	1877
4	Sola3-1N1_AA	DNA/Sola	48093	48376	2271
4	Sola3-1N1_AA	DNA/Sola	62378	62583	1001
4	Sola3-1N1_AA	DNA/Sola	84393	84437	236
2	SPMLIKE	DNA/EnSpm	67313	67448	496
2	SPMLIKE	DNA/EnSpm	67799	67927	335
2	SPMLIKE	DNA/EnSpm	67967	68073	410
2	SPMLIKE	DNA/EnSpm	68353	68487	373
2	SPMLIKE	DNA/EnSpm	69141	69269	407
2	SPMLIKE	DNA/EnSpm	69527	69636	320
2	SPMLIKE	DNA/EnSpm	70868	71003	330
2	SPMLIKE	DNA/EnSpm	71146	71249	274
2	SPMLIKE	DNA/EnSpm	82962	83097	450
2	SPMLIKE	DNA/EnSpm	83110	83186	260
3	TART_DV	NonLTR/Jockey	9798	9847	227
2	TguERVK3a_I	ERV/ERV2	85391	85442	207
2	Transib-12_HM	DNA/Transib	218646	218697	212
4	Transib-17_HM	DNA/Transib	50061	50139	229
4	Transib-6_HM	DNA/Transib	68568	68605	248
2	tRNA-Thr-ACY_	Pseudogene/tRNA	88702	88771	312
2	tRNA-Thr-ACY_	Pseudogene/tRNA	229340	229397	222
3	tRNA-Thr-ACY_	Pseudogene/tRNA	36180	36249	293
2	tRNA-Val-GTA	Pseudogene/tRNA	167558	167610	271
2	tRNA-Val-GTA	Pseudogene/tRNA	212465	212519	233
3	tRNA-Val-GTA	Pseudogene/tRNA	36848	36902	280
2	TWIFBIG	DNA/hAT	210355	210424	217
2	TWIN	NonLTR/SINE	186881	186938	315
3	TWIN	NonLTR/SINE	14594	14652	405
4	TWIN	NonLTR/SINE	45859	45916	362
2	Tx_mos	DNA	183425	183511	272
2	TZF28B	DNA/Mariner	97522	97556	236
2	VANDAL14	DNA/MuDR	143139	143191	211
8	VANDAL22	DNA/MuDR	10335	10396	244
2	VANDAL5	DNA/MuDR	76782	76827	220
2	Waldo1_AG	NonLTR	108545	108754	412
3	Waldo1_AG	NonLTR	10403	10586	470
3	Waldo1_AG	NonLTR	11701	11807	314
8	Waldo1_AG	NonLTR	4153	5367	1605
3	WaldoAg2	NonLTR	12311	12534	565
8	WaldoAg2	NonLTR	2764	2953	387
2	WUJIN	DNA	205808	205962	632
4	WUJIN	DNA	51460	51524	408
2	WUKONG	DNA	48321	48375	318
2	WUKONG	DNA	48600	48820	1261
3	Zator-1_AA	DNA/Zator	37100	37288	1212

4	Zator-1_AA	DNA/Zator	58324	58527	800
2	Zator-1_CP	DNA/Zator	17241	17342	269
2	Zator-1_CP	DNA/Zator	17447	17545	267
2	Zator-1_CP	DNA/Zator	100284	100483	571
2	Zator-1_CP	DNA/Zator	131955	132107	335
2	Zator-1_CP	DNA/Zator	171545	171585	236
2	Zator-1_CP	DNA/Zator	211479	211519	254
2	Zator-1_CP	DNA/Zator	211953	212115	624
2	Zator-1_CP	DNA/Zator	263057	263183	263
3	Zator-1_CP	DNA/Zator	24824	24931	308
3	Zator-1_CP	DNA/Zator	25057	25158	269
3	Zator-1_CP	DNA/Zator	36422	36543	438
4	Zator-1_CP	DNA/Zator	61984	62144	610
4	Zator-1_CP	DNA/Zator	69654	69815	409
2	Zator-1_HM	DNA/Zator	108887	108947	237
2	Zator-2_AA	DNA/Zator	187422	187522	276
4	Zator-2_AA	DNA/Zator	75034	75096	273
2	Zator-2_HM	DNA/Zator	108782	108849	230
2	ZEBEDEE	LTR/Copia	102322	102729	2743
2	ZEBEDEE	LTR/Copia	246459	247009	3678
4	ZEBEDEE	LTR/Copia	36421	36655	1635
2	ZEON2_ZM_I	LTR/Gypsy	46045	46157	297
2	ZEON2_ZM_I	LTR/Gypsy	69328	69392	236
2	ZhAT-N11_ZM	DNA/hAT	84378	84491	233
2	ZhAT-N15_ZM	DNA/hAT	105273	105374	229
