# Supplementary material for: Genomic organization and splicing evolution of the doublesex gene, a Drosophila regulator of sexual differentiation, in the dengue and yellow fever mosquito Aedes aegypti
Source: BMC Evol Biol. 2011 Feb 10;11:41. doi: 10.1186/1471-2148-11-41 (PMC3045327; doi:10.1186/1471-2148-11-41)
Supplement: Additional file 2 — Figure S1 Censor analysis on Aeadsx introns. Censor software graphically maps detected repeats with color-coding of different types of repeats. Legend of Censor output is available at: http://www.girinst.org/censor/help.html#MAP. [file 1471-2148-11-41-S2.PDF]

Additional file 2

Figure S1 - Censor analysis on *Aeadsx* introns

<u>Intron 2</u>

Length: 274879 bp

Censor graphic output:

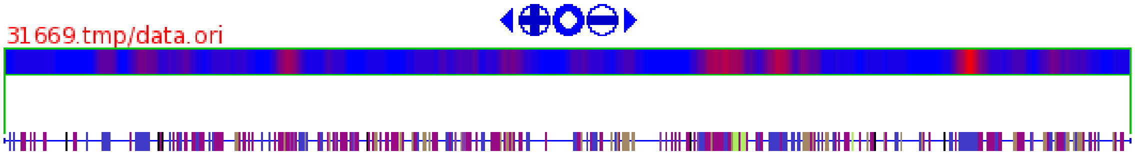

Number of repetitive elements per Kb: 1,14

Percentage of repetitive element nucleotides of intron: 18%

<u>Intron 3</u>

Length: 43797 bp

Censor graphic output:

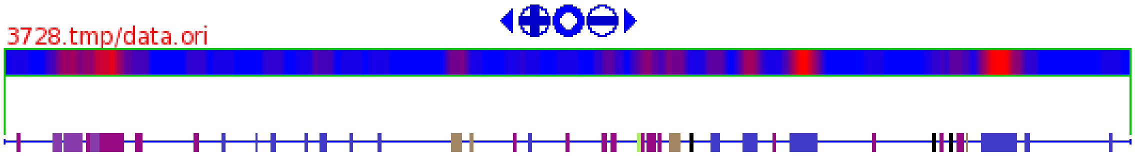

Number of repetitive elements per Kb: 1,27

Percentage of repetitive element nucleotides of intron: 20%

<u>Intron 4</u>

Length: 85670 bp

Censor graphic output:

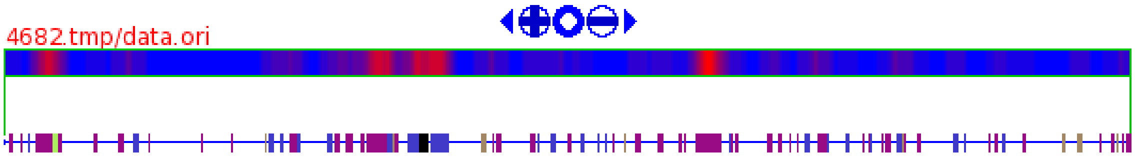

Number of repetitive elements per Kb: 1,12

Percentage of repetitive element nucleotides of intron: 19%

Intron 5

Length: 13860 bp

Censor graphic output:

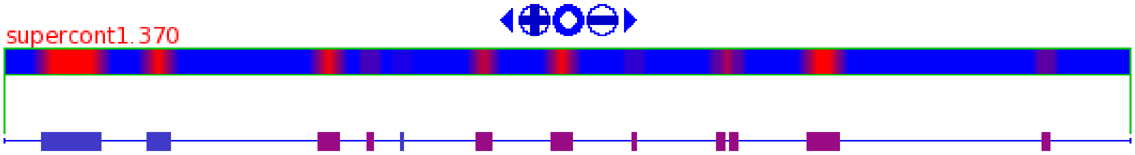

Number of repetitive elements per Kb: 1,0

Percentage of repetitive element nucleotides of intron: 18%

Intron 6

Length: 208 bp

Number of repetitive elements per Kb: 0

Percentage of repetitive element nucleotides of intron: 0%

Intron 7

Length: 10392 bp

Censor graphic output:

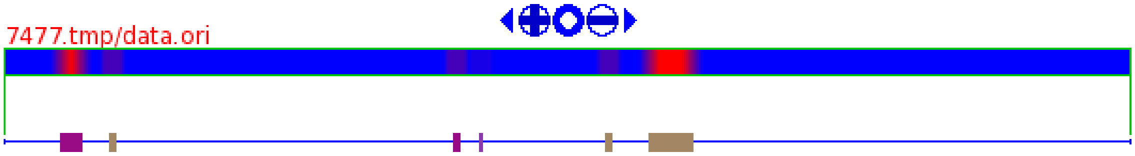

Number of repetitive elements per Kb: 0,6

Percentage of repetitive element nucleotides of intron: 7%

Intron 8

Length: 22437 bp

Censor graphic output:

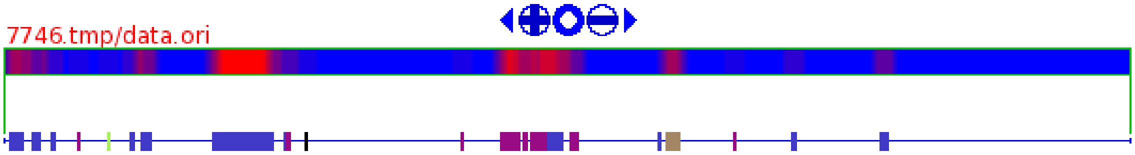

Number of repetitive elements per Kb: 1,2

Percentage of repetitive element nucleotides of intron: 17%
