# Supplementary figures and images for: Genomic organization and splicing evolution of the doublesex gene, a Drosophila regulator of sexual differentiation, in the dengue and yellow fever mosquito Aedes aegypti
Source: BMC Evol Biol. 2011 Feb 10;11:41. doi: 10.1186/1471-2148-11-41 (PMC3045327; doi:10.1186/1471-2148-11-41)

Additional file 3

Figure S2 – Microsynteny analysis

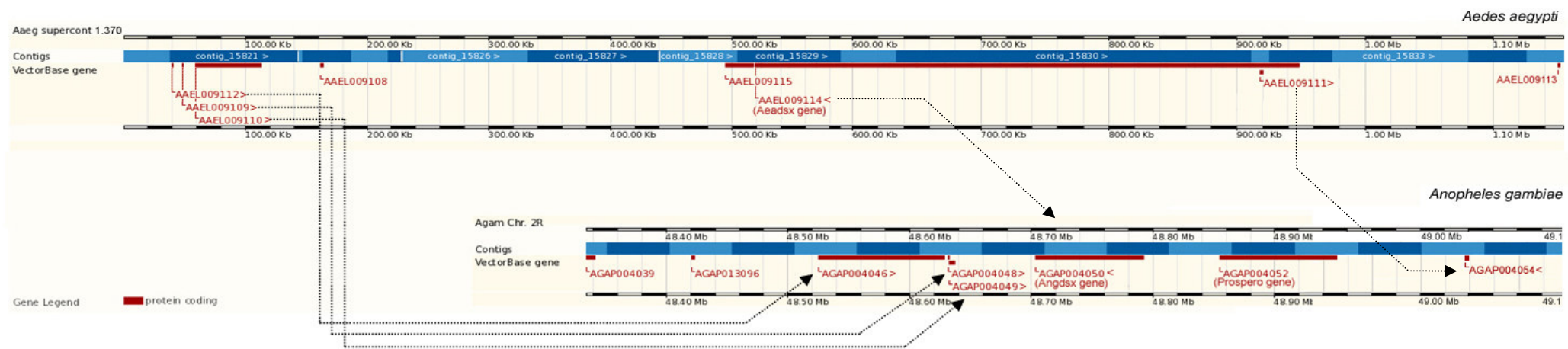

Supplement: Additional file 3 — Figure S3 Microsynteny analysis. Syntenic dsx-containing regions of Aedes aegypti and Anopheles gambiae are reported. In red the position of putative genes. [file 1471-2148-11-41-S3.PDF]
