# Supplementary material for: Genomic organization and splicing evolution of the doublesex gene, a Drosophila regulator of sexual differentiation, in the dengue and yellow fever mosquito Aedes aegypti
Source: BMC Evol Biol. 2011 Feb 10;11:41. doi: 10.1186/1471-2148-11-41 (PMC3045327; doi:10.1186/1471-2148-11-41)
Supplement: Additional file 4 — Tables S2 Putative cis-acting elements identified in Aeadsx and homologous elements in D. melanogaster, An. gambiae and N. vitripennis. Bold letters indicate identical positions respect to the sequences of Drosophila or Nasonia elements. [file 1471-2148-11-41-S4.PDF]

Table S2 – Putative *cis*-acting elements identified in *Aeadsx* and homologue elements in *D. melanogaster*, *An. gambiae* and *N. vitripennis*

Gene		TRA/TRA-2 Binding Site	Identity
Dmdsx		TCTTCAATCAACA<br>AA	
Angdsx	1	TCGCCGATCAACC	9/13
	2	CCATCGTTCAACC	9/13
	3	TCTCCAATCAATC	10/13
	4	ACATCAATCAATA	11/13
	5	ACATCAATCAATC	10/13
Aeadsx	1	AATACAAACAACA	11/13
	2	TCAACAAGCAACA	11/13
	3	TCTTCAACCAACC	11/13
	4	CCTACAATCTACA	11/13
	5	GCTGCAATCAACA	11/13

Gene		PRE	N° of Pur.
Dmdsx		AAAGGACAAAGGACAAAA	16/18
Angdsx	1	CGAGAAAAGGGGAGAGCAAA	18/20
	2	ACAAACGAGAGCAAGGAAAA	17/20
Aeadsx	1	AGAGGGAAAATTTAAAAAATGCAAAA	21/26
	2	GAAAAAATCCGGGAAACCGG	15/20

Gene		*Nasonia vitripennis* TRA/TRA-2 binding motif	Identity
Nvdsx and Nvfru		TGAAGATT<br>G A<br>C C	
Aeadsx	1	CGAAGATC	8/8
	2	GGAAGAAG	6/8
	3	AGAAGAAT	6/8
	4	CGAAGAAA	7/8
	5	AGAAGAAT	6/8

Gene		TRA-2-ISS	Identity
Dmtra-2		CAAGG<br>A	
Aeadsx	1	CAAGA	5/5
	2	CAAGA	5/5
	3	CAAGA	5/5
	4	CAAGA	5/5
	5	CAAGA	5/5
	6	CAAGA	5/5
	7	CAAGG	5/5
	8	CAAGA	5/5

Gene		RBP1 - Type B	Identity
Dmtra-2		ATCCNNA<br>T	
Aeadsx	1	ATCCACA	7/7
	2	ATCTCTA	7/7
	3	ATCCGAA	7/7
	4	ATCTGAA	7/7
	5	ATCTAGA	7/7
	6	ATCCACA	7/7
	7	ATCTACA	7/7
